# Pentapartite Entanglement Measures of GHZ and W-Class State in the Noninertial Frame

**DOI:** 10.3390/e24060754

**Published:** 2022-05-26

**Authors:** Juan Luis Manríquez Zepeda, Juvenal Rueda Paz, Manuel Avila Aoki, Shi-Hai Dong

**Affiliations:** 1 Centro Universitario UAEM Valle de Chalco, Universidad Autónoma del Estado de México, Ecatepec de Morelos 56615, Mexico; buzonjl@gmail.com (J.L.M.Z.); juvenal.rueda@gmail.com (J.R.P.); manvlk@hotmail.com (M.A.A.); 2 Research Center for Quantum Physics, Huzhou University, Huzhou 313000, China; 3 Laboratorio de Información Cuántica, CIDETEC, Instituto Politécnico Nacional, UPALM, Mexico City 07700, Mexico

**Keywords:** GHZ and W-class states, negativity, von Neumann entropy, noninertial frame, 03. 67. a, 03. 67. Mn, 03. 65. Ud, 04. 70. Dy

## Abstract

We study both pentapartite GHZ and W-class states in the noninertial frame and explore their entanglement properties by carrying out the negativities including 1-4, 2-3, and 1-1 tangles, the whole entanglement measures such as algebraic and geometric averages $\pi_{5}$ and $\Pi_{5}$, and von Neumann entropy. We illustrate graphically the difference between the pentapartite GHZ and W-class states. We find that all 1-4, 2-3 tangles and the whole entanglements, which are observer dependent, degrade more quickly as the number of accelerated qubits increases. The entanglements of these quantities still exist even at the infinite acceleration limit. We also notice that all 1-1 tangles of pentapartite GHZ state $N_{\alpha \beta }=N_{\alpha_{I}\beta }=N_{\alpha_{I}\beta_{I}}=0$ where α,β∈(A,B,C,D,E), whereas all 1-1 tangles of the W-class state $N_{\alpha \beta },N_{\alpha_{I}\beta }$ and $N_{\alpha_{I}\beta_{I}}$ are unequal to zero, e.g., $N_{\alpha \beta }=0.12111$ but $N_{\alpha_{I}\beta }$ and $N_{\alpha_{I}\beta_{I}}$ disappear at r>0.61548 and r>0.38671, respectively. We notice that the entanglement of the pentapartite GHZ and W-class quantum systems decays faster as the number of accelerated particles increases. Moreover, we also illustrate the difference of von Neumann entropy between them and find that the entropy in the pentapartite W-class state is greater than that of GHZ state. The von Neumann entropy in the pentapartite case is more unstable than those of tripartite and tetrapartite subsystems in the noninertial frame.

## 1. Introduction

The transfer of quantum states between distant nodes of a quantum network is a basic task for quantum information processing. It is well known that all protocols used for quantum state transmission require entanglement between the sender and the receiver systems. Entanglement, which is at the basis of quantum mechanics and almost every quantum information protocol, has become a very interesting topic, particularly in many-body systems, with the recent development of quantum information technology. The correct understanding of entanglement is of importance due to its special application in many branches such as quantum teleportation, quantum communication and quantum cryptography [1,2,3,4,5,6,7,8,9,10], and quantum algorithms [11,12]. Moreover, it is also helpful in studying quantum communication protocols like quantum key distribution (QKD) [13].

Up to now, the development of quantum computing has required the study of multi-qubit entangled systems, so the entanglement properties of multipartite quantum systems under inertial frames are becoming more and more important. However, with the development of relativistic quantum information science, many authors have paid more and more attention to the development of this field. In order to study the property of quantum entangled state existing in the noninertial frame, we have to employ a relativistic setting [14,15,16]. The relativistic quantum information regarded as a new interesting field has emerged for many years since the relation between quantum information science and relativity theory intermediated by quantum field theory allows us to have a deeper understanding of the fundamental principles in quantum mechanics [17,18]. This also helps us explore how the degree of entanglement would be affected in curved space time, particularly by the acceleration parameter. Until now, quantum information theory has been enriched by the contributions of quantum entanglement made in the noninertial frame [19,20,21]. The properties of multipartite entangled systems are mainly related to the acceleration parameter and the number of particles in the noninertial system, which undoubtedly affect the entanglement degree of the entangled system.

In recent years, many relevant and significant contributions to this field have been made [1,3,14,15,16,22,23,24,25,26,27,28,29,30,31,32,33,34,35]. For example, since several pure multipartite entangled systems was studied [25], where the Unruh effect was discussed [19,20,21], the entanglement has been verified as an observer dependent in the noninertial frame. Compared with the well-known entangled stated-GHZ state [15,36,37,38,39,40,41], the authors paid less attention to the W-class state because its density matrix cannot be written as an X matrix form. Nevertheless, we have employed a special technique to study the density matrix in the non-X matrix form and carried out the tripartite and tetrapartite W-class state cases [42,43,44], except for the generalized GHZ state in the noninertial frame [45,46,47,48]. After studying, we find that the degree of entanglement of the W-class state is more robust than the GHZ and generalized GHZ states. It should be recognized that the entanglement for tripartite and tetrapartite systems still exists even at the infinite acceleration limit. However, Ye and her collaborators carried out the enhancement of multipartite entanglement in an open system in the noninertial frame [49]. Other relevant studies such as the fidelity loss and coherence loss, etc., in the open systems have also been done in Refs. [8,10].

Generally speaking, two main entanglement measures, which are named as negativity and von Neumann entropy, have been used to quantify the genuine entanglement. The negativity is employed to study the multi-tangle and the whole entanglement measures. The von Neumann entropy is concerned with the stability of the entangled system. For example, the three tangle, i.e., 1-2 tangle [50], was proposed to study the entanglement property of the entangled quantum system when tripartite Alice, Bob, and Charlie initially shared an arbitrary fermionic three-qubit pure state as well as the later proposed π-tangle [36]. The three tangle has interesting properties, but its analytical calculation becomes a nontrivial task because its calculation depends on the negativities of bipartite and tripartite systems. Similarly, four tangle has been proposed when we studied the tetrapartite systems, which include 1-3 and 2-2 tangles [42,44,45,47,48]. Recently, stimulated by the study of the tripartite and tetrapartite W-class state [42,43,44], Sun and her coauthors studied the entanglement property of a pentapartite W-class state in the noninertial frame and showed how the acceleration parameter and the number of the accelerated qubits affect the entanglement property of the pentapartite W-class entangled system [51]. This was realized by studying the π-tangle, including the 1-4, 1-1 tangles and the von Neumann entropy. However, they were not concerned with the 2-3 tangle case due to its complication, which is also an important factor to describe the entanglement property of the entangled system. Moreover, as the W-class and GHZ states are the two most important pure states in quantum information, it is necessary to study their entanglement properties simultaneously and show their difference graphically. To enrich the paper [51], we shall present all 1-4, 2-3 tangles and von Neumann entropy for these two important pure states for completeness, which is the main purpose of this work.

This paper is organized as follows. In Section 2, we briefly review the transformation between Minkowski space and Rindler coordinates. How to construct the density matrix for the simplest case is presented. In Section 3, we study the negativities, including 1-4, 2-3, and 1-1 tangles, whole entanglement measures $\pi_{5}$ and $\Pi_{5}$ and von Neumann entropy, which are illustrated graphically to show their difference. Finally, in Section 4 we summarize our conclusions.

## 2. Pentapartite Entanglement from One to Five Accelerated Observers

The pentapartite GHZ state that we are going to study in this work is given by

(1) $$|\mathrm{GHZ}\rangle =\frac{1}{\sqrt{2}}\left(|0_{A}0_{B}0_{C}0_{D}0_{E}\rangle +|1_{A}1_{B}1_{C}1_{D}1_{E}\rangle \right),$$

where $|0_{A}0_{B}0_{C}0_{D}0_{E}\rangle =|0_{A}\rangle ⊗|0_{B}\rangle ⊗|0_{C}\rangle ⊗|0_{D}\rangle ⊗|0_{E}\rangle$ so does the state $|1_{A}1_{B}1_{C}1_{D}1_{E}\rangle$, while the pentapartite W-class state has the following form [52]:

(2) $$|W\rangle =\frac{1}{\sqrt{5}}\left(|0_{A}0_{B}0_{C}0_{D}1_{E}\rangle +|0_{A}0_{B}0_{C}1_{D}0_{E}\rangle +|0_{A}0_{B}1_{C}0_{D}0_{E}\rangle +|0_{A}1_{B}0_{C}0_{D}0_{E}\rangle +|1_{A}0_{B}0_{C}0_{D}0_{E}\rangle \right).$$

Here we use the subscripts A, B, C, D, and E to denote Alice, Bob, Charlie, David, and Elly (as we know, Eve is not of confidence), respectively. They initially share a pentapartite GHZ or W-class state in the inertial frame. In this work, we assume that the acceleration of particles always starts from the rightmost one in A(Alice), B(Bob), C(Charlie), D(David), E(Elly) qubits. That is to say, we first suppose that Elly is accelerated in a uniform acceleration but Alice, Bob, Charlie, and David remain stationary, and so on.

**Figure 1:**
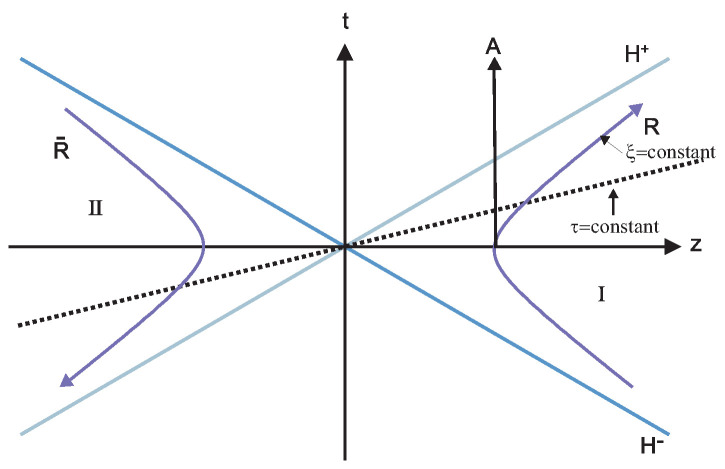
Rindler space time diagram: lines of constant position ξ are hyperbolas and lines of constant proper time τ for the accelerated observer run through the origin. In present work, we denote regions I and II as Bob and anti-Bob, respectively. The reader can refer to Ref. [55] for more information.

For entangled GHZ and W-Class states in the noninertial frame, let us use Rindler coordinates to describe a family of observers with a uniform acceleration and divide Minkowski space-time into two inaccessible regions I and II. The rightward accelerating observers are located in region I and causally disconnected from the analogous counterparts in region II [53,54]. Let us briefly review the connection between the vacuum and excitation states in Minkowski coordinates and those in Rindler coordinates. First, let Alice stay stationary, while Bob moves in a uniform acceleration. We consider Bob accelerated uniformly in the (t,z) plane. Rindler coordinates (τ,ζ) are appropriate for describing the viewpoint of an observer moving in a uniform acceleration. Two different sets of Rindler coordinates, which differ from each other by an overall change in sign, are necessary for covering Minkowski space. These sets of coordinates define two Rindler regions disconnected from each other, as shown in Figure 1 [16,55]:

(3) $$\begin{array}{c} t=a^{-1}e^{a\zeta }\sinh\left(a\tau \right),\;z=a^{-1}e^{a\;\zeta }\cosh\left(a\tau \right),\;\mathrm{Region}\;I,\\ t=-a^{-1}e^{a\zeta }\sinh\left(a\tau \right),\;z=-a^{-1}e^{a\;\zeta }\cosh\left(a\tau \right),\;\mathrm{Region}\;\mathrm{II}. \end{array}$$

A free Dirac field in (3+1) dimensional Minkowski space satisfies the Dirac equation $i\gamma^{\mu }\partial_{\mu }\psi -m\psi =0$, where *m* is the particle mass and $\gamma^{\mu }$ the Dirac gamma matrices. A spinor wave function ψ composed of the complete orthogonal set of fermion $\psi_{k}^{+}$ and antifermion $\psi_{k}^{-}$ modes can be expressed as $\psi =\int (a_{k}\psi_{k}^{+}+b_{k}^{†}\psi_{k}^{-})dk$, where $a_{k}^{†}\left(b_{k}^{†}\right)$ and $a_{k}\left(b_{k}\right)$ are the creation and annihilation operators for fermions (antifermions) of the momentum *k*, respectively, satisfying the relation $\left\{a_{i},a_{j}^{†}\right\}=\left\{b_{i},b_{j}^{†}\right\}=\delta_{ij}$. The quantum field theory for a Rindler observer is constructed by expanding the spinor field in light of a complete set of fermion and antifermion modes in regions I and II as follows:

(4) $$\psi =\int \sum_{\tau }\left(c_{k}^{\tau }\psi_{k}^{\tau +}+d_{k}^{\tau †}\psi_{k}^{\tau -}\right)dk,\;\;\;\;\tau \in \left\{I,\mathrm{II}\right\}.$$

In a similar way, $c_{k}^{\tau †}\left(d_{k}^{\tau †}\right)$ and $c_{k}^{\tau }\left(d_{k}^{\tau }\right)$ are the creation and annihilation operators for fermion (antifermions), respectively, acting on region I (II) for τ=I(II) and also satisfy a similar anticommutation relation. The relation between creation and annihilation operators in Minkowski and Rindler space times can be found by the Bogoliubov transformation

(5) $$a_{k}=\cos\left(r\right)\;c_{k}^{I}-\sin\left(r\right)\;d_{-k}^{\mathrm{II}†},b_{k}=\cos\left(r\right)\;d_{k}^{I}-\sin\left(r\right)\;c_{-k}^{\mathrm{II}†},$$

where $\cos\left(r\right)=1/\sqrt{1+e^{-2\pi \omega_{k}c/a}}$ with $\omega_{k}=\sqrt{{\left|k\right|}^{2}+m^{2}}$ and *r* is a Bob’s acceleration parameter with the range r∈[0,π/4] for a∈[0,∞). It is known from this equation and its adjoint that Bogoliubov transformation mixes a fermion in region I and antifermions in region II. As a result, it is assumed that the Minkowski particle vacuum state for mode *k* based on Rindler Fock states is given by

(6) $$|0_{k}\rangle_{M}=\sum_{n=0}^{1}A_{n}|n_{k}\rangle_{I}^{+}{|n_{-k}\rangle }_{II}^{-},$$

where the Rindler region I or II Fock states carry a subscript I and II, respectively, on the kets, but the Minkowski Fock states are indicated by the subscript *M* on the kets. As what follows, we are only interested in using single mode approximation [15,16,24,56,57,58,59], i.e., $w_{A,B,C,D}=w$ and also uniform acceleration $a_{A,B,C,D}=a$ ($a_{w,M}\approx a_{w,U}$ is considered to relate Minkowski and Unruh modes) for simplicity.

Using the single mode approximation, one can transform Bob’s vacuum state $|0_{B}\rangle_{M}$ and one-particle state $|1_{B}\rangle_{M}$ in Minkowski space into Rindler space. Using the creation and annihilation operators on Equation (6) above and using the normalization condition, we can obtain [15,16,24,56,57,58,59]

(7) $$\begin{array}{c} {|0\rangle }_{M}=\cos\left(r\right)|0_{I}0_{II}\rangle +\sin\left(r\right)|1_{I}1_{II}\rangle ,\\ {|1\rangle }_{M}=|1_{I}0_{II}\rangle , \end{array}$$

where $|n_{B_{I}}\rangle$ and $|n_{B_{II}}\rangle$ (n=0,1) are the mode decomposition of $|n_{B}\rangle$ into two causally disconnected regions I and II in Rindler space. It should be pointed out that Bruschi et al. discussed the Unruh effect *beyond* the single mode approximation [21], in which two complex numbers $q_{R}$ and $q_{L}$ (the subindexes *L* and *R* corresponding to the Left and Right regions in Rindler diagram, i.e., regions I and II) are used to construct the one-particle state, i.e., $|1\rangle =q_{R}|1_{R}0_{L}\rangle +q_{L}|0_{R}1_{L}\rangle$. However, in the present case for single mode approximation, one has $q_{R}=1,q_{L}=0$ to satisfy the normalization condition $|q_{R}{|}^{2}+{\left|q_{L}\right|}^{2}=1$. It is also worth noting that a Minkowski mode that defines the Minkowski vacuum is related to a highly nonmonochromatic Rindler mode rather than a single mode with the same frequency (see Refs. [21,30,60,61] for details). Other relevant contributions [31,59,62,63,64,65] have also been made.

To illustrate how to expand $|\mathrm{GHZ}\rangle$ in Rindler coordinates, we are going to give explicit expression when Elly is accelerated, i.e.,

(8) $${|\mathrm{GHZ}\rangle }_{ABCDE_{I}E_{\mathrm{II}}}=\frac{1}{\sqrt{2}}\left(\cos\left(r\right)|0_{A}0_{B}0_{C}0_{D}0_{E_{I}}0_{E_{\mathrm{II}}}\rangle +\sin\left(r\right)|0_{A}0_{B}0_{C}0_{D}1_{E_{I}}1_{E_{\mathrm{II}}}\rangle +|1_{A}1_{B}1_{C}1_{D}1_{E_{I}}0_{E_{\mathrm{II}}}\rangle \right).$$

Similarly, we can also obtain the expressions of other cases when the observers Alice, Bob, Charlie, and David are accelerated. Such a procedure also works for the pentapartite W-class $|W\rangle$ case.

After the transformation to the Rindler space, we have to trace out the part of the antiparticle state in region II from the density matrix $\rho_{ABCDE_{I}}={\mathrm{Tr}}_{E_{II}}{|\mathrm{GHZ}\rangle }_{ABCDE_{I}E_{\mathrm{II}}}\langle \mathrm{GHZ}|$. In this case, when Elly is accelerated, the corresponding density matrix is thus given by

(9) $$\begin{array}{c} \rho_{ABCDE_{I}}=\frac{1}{2}(\cos^{2}\left(r\right)|0_{A}0_{B}0_{C}0_{D}0_{E_{1}}\rangle \langle 0_{A}0_{B}0_{C}0_{D}0_{E_{I}}|+\cos\left(r\right)|0_{A}0_{B}0_{C}0_{D}0_{E_{I}}\rangle \langle 1_{A}1_{B}1_{C}1_{D}1_{E_{I}}|\\ \;\;\;\;\;\;\;\;\;\;\;\;\;\;\;\;+\cos^{2}\left(r\right)|0_{A}0_{B}0_{C}0_{D}1_{E_{I}}\rangle \langle 0_{A}0_{B}0_{C}0_{D}1_{E_{I}}|+\cos\left(r\right)|0_{A}0_{B}0_{C}0_{D}0_{E_{I}}\rangle \langle 1_{A}1_{B}1_{C}1_{D}1_{E_{I}}|\\ \;\;\;\;\;\;\;\;\;\;\;\;\;\;\;\;+|1_{A}1_{B}1_{C}1_{D}1_{E_{I}}\rangle \langle 1_{A}1_{B}1_{C}1_{D}1_{E_{I}}|). \end{array}$$

For simplicity, we write out explicitly all nonzero elements [i,j] for pentapartite GHZ and W-class states in Appendix A. These results will be helpful in calculating the negativity and von Neumann entropy, as shown below.

## 3. Entanglement Measures: Negativity and von Neumann Entropy

### 3.1. Negativity

Negativity, which is used to measure the entanglement of multipartite systems, is defined by [66,67,68]

(10) $$\begin{array}{c} N_{\alpha ,\beta \gamma \delta \epsilon }=\left|\right|\rho_{\alpha ,\beta \gamma \delta \epsilon }^{T_{\alpha }}\left|\right|-1,\;\;\;N_{\alpha \beta ,\gamma \delta \epsilon }=\left|\right|\rho_{\alpha \beta (\gamma \delta \epsilon )}^{T_{\alpha \beta }}\left|\right|-1,\;\;\;N_{\alpha ,\beta }=\left|\right|\rho_{\alpha \beta }^{T_{\alpha }}\left|\right|-1, \end{array}$$

where $N_{\alpha ,\beta \gamma \delta \epsilon }$, $N_{\alpha \beta ,\gamma \delta \epsilon }$ and $N_{\alpha ,\beta }$ represent 1-4, 2-3, and 1-1 tangles, respectively. The expressions $\left|\right|\rho_{\alpha (\beta \gamma \delta \epsilon )}^{T_{\alpha }}\left|\right|$, $\left|\right|\rho_{\alpha \beta (\gamma \delta \epsilon )}^{T_{\alpha \beta }}\left|\right|$ and $\left|\right|\rho_{\alpha \beta }^{T_{\alpha }}\left|\right|$ are the trace norms of the partial transposes of the density matrices. Generally speaking, the trace of any Hermitian operator *A* is equal to the sum of its eigenvalues [69], $\left||A|\right|=\mathrm{tr}\sqrt{A^{†}A}$, i.e.,

(11) $$\begin{array}{c} \left||M|\right|-1=2\sum_{i=1}^{N}{\left|\lambda_{M}^{\left(-\right)}\right|}^{i}, \end{array}$$

where $\lambda_{M}^{\left(-\right)}$ represents the negative eigenvalue of the matrix *M*. It should be pointed out the calculation of these negativities is very complicated and time consuming.

**Figure 2:**
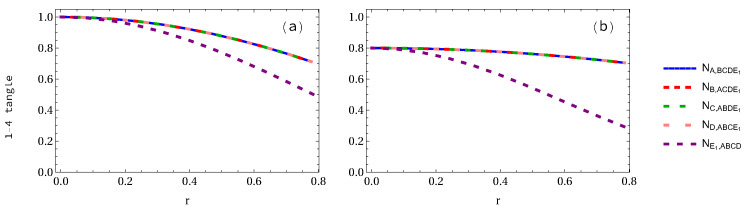
Panels (**a**,**b**) show the variation of 1-4 tangle with the parameter *r* in the case of pentapartite GHZ and W-class states, respectively, when only one qubit is accelerated.

Let us first calculate the negativity 1-4 tangle for the GHZ and W-class states when 1 to 5 observer(s) is (are) accelerated. The explicit expressions of the pentapartite GHZ and W-class states are written out in Appendix B for completeness. (It should be pointed out that the special symbols such as Root, #, and & appeared in this Appendix B and also in Appendix C are generated systematically by Wolfram Mathematica.) In Figure 2, we plot the negativity 1-4 tangle of pentapartite GHZ and W-class states when only one of five observers is accelerated. It is seen in Figure 2a that the $N_{A,BCDE_{1}}=N_{B,ACDE_{1}}=N_{C,ABDE_{1}}=N_{D,ABCE_{1}}$ decreases from 1 to $1/\sqrt{2}$ (29.29% loss of entanglement), whereas the $N_{E_{1},ABCD}$ decreases from 1 to 0.5 (50% loss of entanglement). This means that entanglement is observer dependent. Furthermore, in the case of the W-class state as shown in Figure 2b, the $N_{A,BCDE_{1}}$ decreases from 0.8 to 0.7048 (11.891% loss of entanglement), but $N_{E_{1},ABCD}$ decreases from 0.8 to 0.29282 (63.4% loss of entanglement).

**Figure 3:**
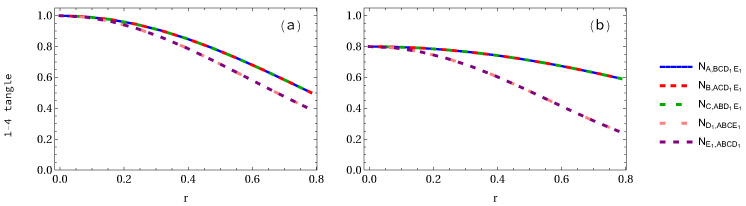
Same as Figure 2 but when two qubits are accelerated.

In Figure 3, we plot the negativity 1-4 tangle when two observers are accelerated. As shown in Figure 3a, the negativity 1-4 tangle $N_{A,BCD_{1}E_{1}}=N_{B,ACD_{1}E_{1}}=N_{C,ABD_{1}E_{1}}$ in the pentapartite GHZ system decreases from 1 to 0.5 (50% loss of entanglement), whereas $N_{E_{1},ABCD_{1}}=N_{D_{1},ABCE_{1}}$ decreases to 0.3903 (60.97% loss of entanglement). In the case of the W-class state as displayed in Figure 3b, the negativity $N_{A,BCD_{1}E_{1}}$ decreases from 0.8 to 0.5924 (25.94% loss of entanglement), whereas the $N_{E_{1},ABCD}$ decreases from 0.8 to 0.24515 (81.86% loss of entanglement). We may conclude that the negativity $N_{A,BCD_{1}E_{1}}$ in the GHZ state decays faster than that of W-class state, but $N_{E_{1},ABCD_{1}}$ in the GHZ state decays slower than that of W-class state.

**Figure 4:**
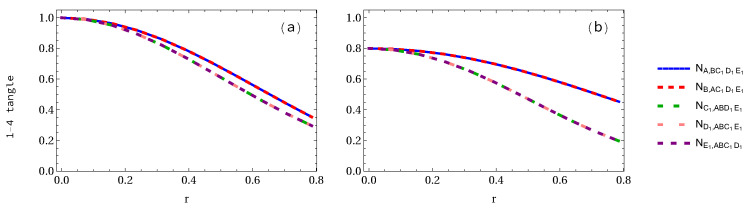
Same as Figure 2 and Figure 3 but when three qubits are accelerated.

In Figure 4, we plot the negativity 1-4 tangle when three observers are accelerated. We can see in Figure 4a that negativity $N_{A,BC_{1}D_{1}E_{1}}=N_{B,AC_{1}D_{1}E_{1}}$ in the GHZ pentapartite system decreases from 1 to $1/2\sqrt{2}$ (64.65% loss of entanglement), and $N_{E_{1},ABC_{1}D_{1}}=N_{C_{1},ABD_{1}E_{1}}=N_{D_{1},ABC_{1}E_{1}}$ decreases to 0.2965 (70.35% loss of entanglement). However, in the case of the W-class state as shown in Figure 4b, $N_{A,BC_{1}D_{1}E_{1}}$ decreases from 0.8 to 0.4529 (43.38% loss of entanglement), whereas $N_{E_{1},ABC_{1}D_{1}}$ decreases to 0.1966 (75.41% loss of entanglement).

**Figure 5:**
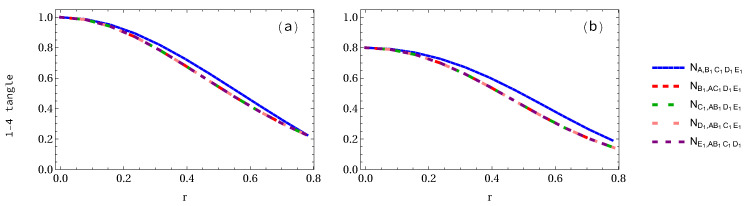
Same as above but when four qubits are accelerated.

In Figure 5, we plot the negativity 1-4 tangle when four observers are accelerated. We can see in Figure 5a that negativity $N_{A,B_{1}C_{1}D_{1}E_{1}}$ in the GHZ pentapartite state decreases from 1 to 0.2206 (77.94% loss of entanglement), and $N_{E_{1},AB_{1}C_{1}D_{1}}=N_{D_{1},AB_{1}C_{1}E_{1}}=N_{C_{1},AB_{1}D_{1}E_{1}}=N_{B_{1},AC_{1}D_{1}E_{1}}$ decreases also from 1 to 0.2206 (77.94% loss of entanglement). It should be emphasized that the negativities $N_{A,B_{1}C_{1}D_{1}E_{1}}$ and $N_{E_{1},AB_{1}C_{1}D_{1}}$ at both r=0 and r=π/4 are the same, but their explicit expressions given in Appendix B are not the same. Moreover, for the W-class state as shown in Figure 5b, the $N_{A,B_{1}C_{1}D_{1}E_{1}}$ decreases from 0.8 to 0.1870 (76.62% loss of entanglement), but $N_{E_{1},AB_{1}C_{1}D_{1}}$ decreases to a smaller value 0.1436 (82.04% loss of entanglement). We find that the difference of the loss of entanglement for both $N_{E_{1},AB_{1}C_{1}D_{1}}$ and $N_{A,B_{1}C_{1}D_{1}E_{1}}$ of two different pentapartite states is very small.

**Figure 6:**
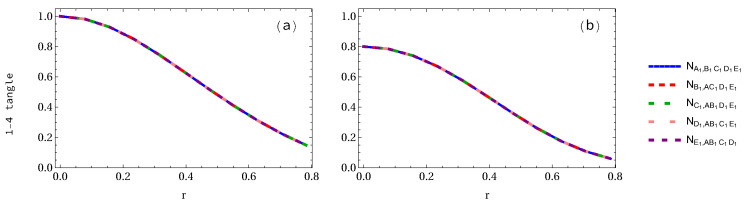
Same as above but when all qubits are accelerated.

Finally, we find that negativity $N_{A_{1},B_{1}C_{1}D_{1}E_{1}}$ in the case of GHZ state decreases from 1 to 0.1455 (85.45% loss of entanglement) as seen in Figure 6a, whereas $N_{A,B_{1}C_{1}D_{1}E_{1}}$ as shown in Figure 6b in the W-class state decreases from 0.8 to 0.0596 (92.54% loss of entanglement). This means that the entanglement of these two pentapartite quantum systems decay most when all particles are accelerated.

**Figure 7:**
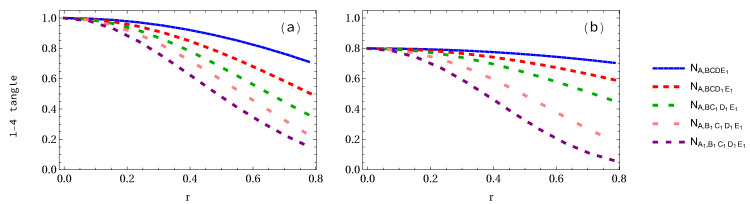
Panels (**a**,**b**) corresponding to GHZ and W-class states with respect to Alice show the variations of the 1-4 tangle for 1 to 5 arbitrary selected qubits as a function of the acceleration parameter *r*.

**Figure 8:**
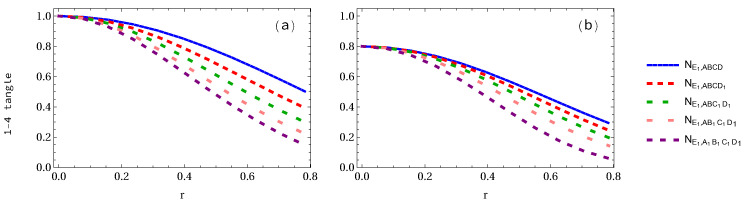
Same as Figure 7 but with respect to Elly.

In Figure 7a,b and Figure 8a,b, we show how the entanglement changes with the number of the accelerated qubits if we only refer to Alice and Elly. It is found that the negativity decreases as the number of accelerated qubits increases, but negativity in the W-class state decreases faster than that in the GHZ state when Elly is taken as a reference (see Figure 8).

**Figure 9:**
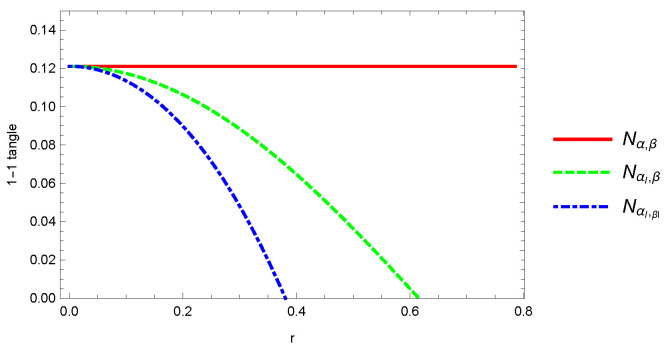
Plot of 1-1 tangle for pentapartite W-class state as a function of acceleration parameter *r*.

To calculate the whole entanglement measures such as algebraic and geometric averages $\pi_{5}$ and $\Pi_{5}$, we have to find the 1-1 tangle of both GHZ and W-class states. In the case of the GHZ state, all 1-1 tangles are equal to zero. In the case of the W-class state, however, some of them that are unequal to zero are expressed as

(12) $$\begin{array}{ccc} N_{\alpha ,\beta } & = & \frac{1}{5}\left(\sqrt{13}-3\right)=0.12111,\\ N_{\alpha_{I},\beta } & = & \frac{1}{5}\left(\sqrt{5+6\cos2r+2\cos4r}-\cos2r-2\right),\\ N_{\alpha_{I},\beta_{I}} & = & \frac{1}{20}\left(4\cos2r-3\cos4r-13+2\sqrt{2}\sqrt{21-12\cos2r+17\cos4r}\right), \end{array}$$

where α,β∈(A,B,C,D,E) and $N_{\alpha ,\beta }>N_{\alpha_{I},\beta }>N_{\alpha_{I},\beta_{I}}>N_{\alpha ,\beta_{1}}=0$. The $N_{\alpha ,\beta }$, $N_{\alpha_{I},\beta }$, and $N_{\alpha_{I},\beta_{I}}$ represent the bipartite subsystems with 0 to 2 accelerated qubits. As shown in Figure 9, it is interesting to see that the entanglement in the 1-1 tangle $N_{\alpha_{I},\beta }$ vanishes at r>0.61548 (only one accelerated particle), but $N_{\alpha_{I},\beta_{I}}$ vanishes at r>0.38671 (two accelerated particles), except for a constant $N_{\alpha ,\beta }=0.12111$.

**Figure 10:**
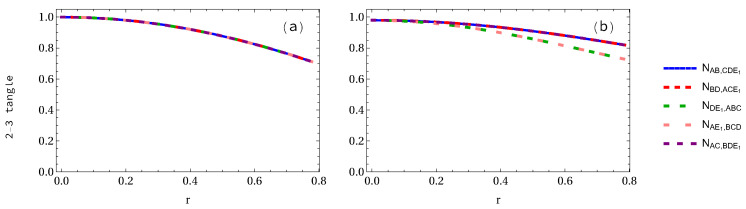
Panels (**a**,**b**) show the 2-3 tangle for both GHZ and W-class states, respectively, when only one qubit is accelerated.

We are now in the position to study negativity 2-3 tangle even though it is not required to calculate the whole entanglement measures. However, we want to present them for completeness, as these results have never been presented to our best knowledge. The analytical expressions of both GHZ and W-class states are given in Appendix C. It is found that the negativity of GHZ state at r=0 is 1, whereas in W-class state it is 0.979796. As the acceleration parameter *r* increases, they all decrease with it, but in W-class state decreases faster than that in the GHZ state. Negativity 2-3 tangle is also dependent on the number of accelerated observers. When only one qubit is accelerated in the GHZ pentapartite state, as displayed in Figure 10a, we notice that $N_{AB,CDE_{1}}=N_{AE_{1},BCD}$ are equal to each other, but $N_{AB,CDE_{1}}$ and $N_{AE_{1},BCD}$ are not the same, as shown in Figure 10b. At the infinite acceleration limit, the 2-3 tangle for GHZ state decreases from 1 to $1/\sqrt{2}$ (29.3% loss of entanglement) at r=π/4, but 2-3 tangle $N_{AB,CDE_{1}}$ and $N_{DE_{1},ABC}$ in the case of W-class state, as shown in Figure 10b, decrease from 0.979796 to 0.8195 (16.35% loss of entanglement) and 0.7278 (25.71% loss of entanglement), respectively.

**Figure 11:**
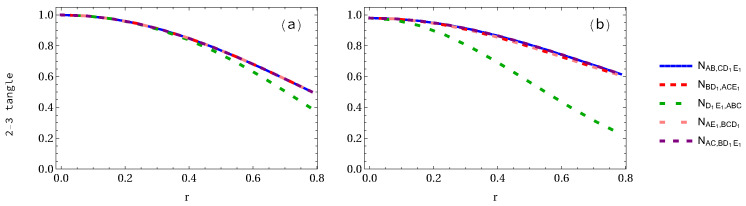
Panels (**a**,**b**) show the 2-3 tangle for both GHZ and W-class states, respectively, when two qubits are accelerated.

When two accelerated observers are considered (see Figure 11), it is found that $N_{D_{1}E_{1},ABC}$ decreases faster than that of $N_{AB,CD_{1}E_{1}}=N_{AE_{1},BCD_{1}}$ in the case of GHZ state. In the W-class case, the difference between $N_{AB,CD_{1}E_{1}}=N_{AC,BD_{1}E_{1}}$ and $N_{AE_{1},BCD_{1}}=N_{BD_{1},ACE_{1}}$ is almost equal to zero, but the difference between $N_{D_{1}E_{1},ABC}$ and others such as $N_{AE_{1},BCD_{1}}$ and $N_{AB,CD_{1}E_{1}}$ is very big. At the infinite acceleration limit, in the case of GHZ state the 2-3 tangles $N_{AB,CD_{1}E_{1}}=N_{BD_{1},ACE_{1}}$ and $N_{D_{1}E_{1},ABC}$ are equal to 0.5 and 0.3903, respectively, but $N_{AB,CD_{1}E_{1}}$ and $N_{D_{1}E_{1},ABC}$ in the case of W-class state are equal to 0.6159 and 0.2274, respectively. This implies that the negativity $N_{AB,CD_{1}E_{1}}$ in the W-class state is bigger than that of GHZ state, whereas $N_{D_{1}E_{1},ABC}$ in the W-class state is smaller than that of GHZ state in the infinite acceleration limit.

**Figure 12:**
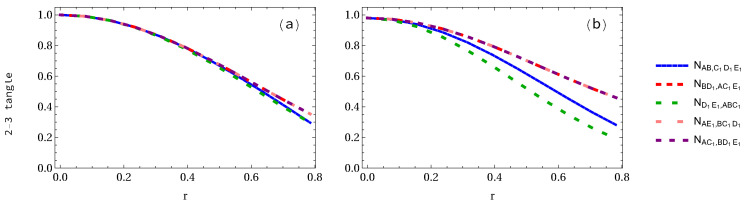
Panels (**a**,**b**) show the 2-3 tangle for both GHZ and W-class states, respectively, when three observers are accelerated.

When three observers are accelerated (see Figure 12), we notice that the difference among $N_{BD_{1},AC_{1}E_{1}}=N_{AE_{1},BC_{1}D_{1}}=N_{AC_{1},BD_{1}E_{1}}$, $N_{AB,C_{1}D_{1}E_{1}}$ and $N_{D_{1}E_{1},ABC_{1}}$ is very small in the GHZ state, as shown in Figure 12a, but in the case of W-class state case, as illustrated in Figure 12b, i.e., their difference is big. In the case of GHZ state when r=π/4, the 2-3 tangle $N_{BD_{1},AC_{1}E_{1}}$ is 0.353553, but $N_{AB,C_{1}D_{1}E_{1}}=N_{D_{1}E_{1},ABC_{1}}$ is equal to 0.2965. However, in the W-class state case, as seen in Figure 12b, we find that their difference is obvious, that is, $N_{BD_{1},AC_{1}E_{1}}=N_{AE_{1},BC_{1}D_{1}}=N_{AC_{1},BD_{1}E_{1}}=0.4566$, $N_{AB,C_{1}D_{1}E_{1}}=0.2774$ and $N_{D_{1}E_{1},ABC_{1}}=0.1873$, respectively.

**Figure 13:**
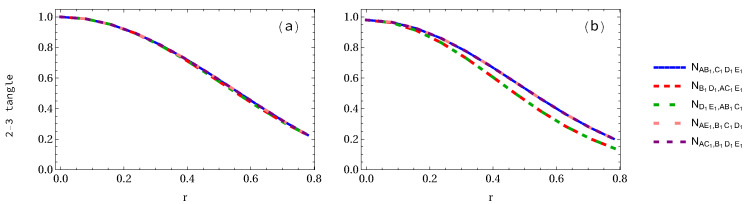
Panels (**a**,**b**) show the 2-3 tangle for both GHZ and W-class states, respectively, when four qubits are accelerated.

When four qubits are accelerated, in the case of GHZ state, as displayed in Figure 13a, we find that the difference between $N_{AB_{1},C_{1}D_{1}E_{1}}=N_{AE_{1},B_{1}C_{1}D_{1}}=N_{AC_{1},B_{1}D_{1}E_{1}}$ and $N_{D_{1}E_{1},AB_{1}C_{1}}=N_{B_{1}D_{1},AC_{1}E_{1}}$ is very small, but in the case of W-class state their difference is a little big. At the infinite limit, the 2-3 tangle is equal to 0.2206 in the case of GHZ state, whereas in the W-class state, the 2-3 tangles $N_{AB_{1},C_{1}D_{1}E_{1}}=0.1976$ and $N_{B_{1}D_{1},AC_{1}E_{1}}=0.1376$, respectively.

**Figure 14:**
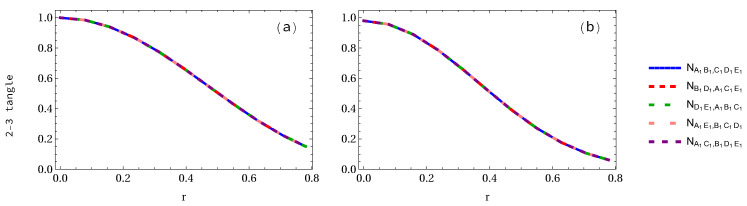
Panels (**a**,**b**) show the 2-3 tangle for both GHZ and W-class states, respectively, when all qubits are accelerated.

**Figure 15:**
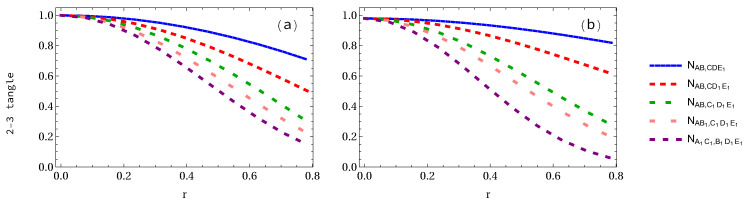
Panels (**a**,**b**) show the 2-3 tangles for both GHZ and W-class states, respectively, when 1 to 5 qubits is (are) accelerated.

Finally, let us consider the case when all the observers are accelerated simultaneously. We see that all 2-3 tangles of either GHZ state or W-class state are equal to each other, as shown in Figure 14. At r=π/4, the 2-3 tangles of GHZ and W-class states are equal to 0.145527 and approximately 0.05, respectively. The variations of the 2-3 tangles for 1 to 5 arbitrary selected accelerated qubits are displayed in Figure 15. It is found that they all decrease with both the increasing acceleration parameter *r* and the number of accelerated qubits.

### 3.2. Whole Entanglement Measures

Now, we use the algebraic average π-tangle to describe the multipartite entanglement defined by [50,70]

(13) $$\begin{array}{c} \pi_{A}=N_{A,BCDE}^{2}-N_{A,B}^{2}-N_{A,C}^{2}-N_{A,D}^{2}-N_{A,E}^{2},\\ \pi_{B}=N_{B,ACDE}^{2}-N_{B,A}^{2}-N_{B,C}^{2}-N_{B,D}^{2}-N_{B,E}^{2},\\ \pi_{C}=N_{C,ABDE}^{2}-N_{C,A}^{2}-N_{C,B}^{2}-N_{C,D}^{2}-N_{C,E}^{2},\\ \pi_{D}=N_{D,ABCE}^{2}-N_{D,A}^{2}-N_{D,B}^{2}-N_{D,C}^{2}-N_{D,E}^{2},\\ \pi_{E}=N_{E,ABCD}^{2}-N_{E,A}^{2}-N_{E,B}^{2}-N_{E,C}^{2}-N_{E,D}^{2}, \end{array}$$

from which we are able to calculate the whole residual entanglement $\pi_{5}$-tangle defined by $\pi_{5}=\left(\pi_{A}+\pi_{B}+\pi_{C}+\pi_{D}+\pi_{E}\right)/5$. Moreover, we may use another whole residual entanglement measure named as geometric average $\Pi_{5}={\left(\pi_{A}\cdot \pi_{B}\cdot \pi_{C}\cdot \pi_{D}\cdot \pi_{E}\right)}^{\frac{1}{5}}$ [71].

**Figure 16:**
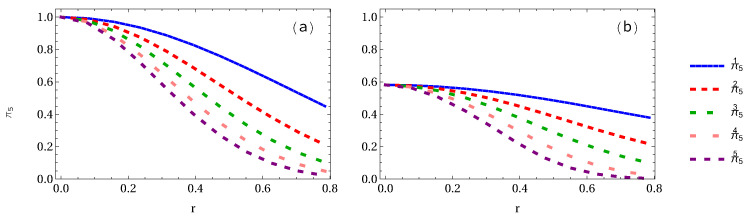
Panels (**a**,**b**) show the whole residual entanglement measure $\pi_{5}$ of GHZ and W-class states, respectively, when 1 to 5 observers is (are) accelerated.

Let us first calculate the whole residual entanglement measures $\pi_{5}$ and $\Pi_{5}$ of both GHZ and W-class states and then plot them. In Figure 16a, it is found that the algebraic average $\pi_{5}$ in the GHZ state decreases from 1 to 0.45, 0.2109, 0.1027, 0.0487, and 0.0211 for 1 to 5 arbitrary selected qubits, respectively. In Figure 16b, we show the whole residual entanglement $\pi_{5}$ in the W-class state. It is interesting to note that $\pi_{5}$ decreases from an initial value 0.5813 to 0.3793 (34.73% loss of entanglement), 0.2170 (62.66% loss of entanglement), 0.0994 (82.90% loss of entanglement), 0.0234 (95.95% loss of entanglement), and 0.0035 (99.38% loss of entanglement), respectively.

**Figure 17:**
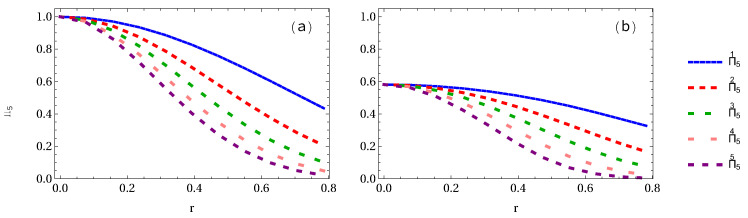
Same as Figure 16 but for the whole entanglement measures $\Pi_{5}$.

**Figure 18:**
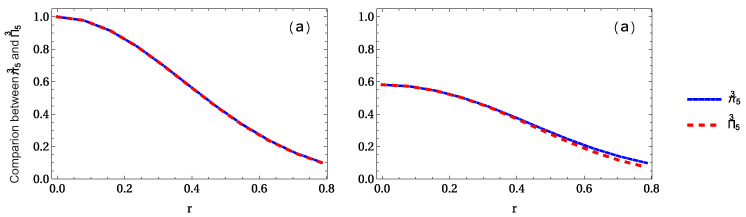
Panels (**a**,**b**) show the difference between whole entanglement measure $\pi_{5}$ when 3 observers are accelerated for the GHZ and W-class states, respectively.

Finally, let us show the variation of the geometric average $\Pi_{5}$ when 1 to 5 accelerated qubits is (are) considered. It is found that $\Pi_{5}$ is very similar to $\pi_{5}$, i.e., whole residual entanglement $\pi_{5}$ and $\Pi_{5}$ are almost same, as shown in Figure 17. To see this clearly, as shown in Figure 18a,b, there is no difference between residual entanglement $\overset{3}{\pi_{5}}$ and $\overset{3}{\Pi_{5}}$ in the GHZ state, but there is a very slight difference in the W-class state.

### 3.3. Entropy

Another useful quantity to measure entanglement is the von Neumann entropy defined by [72,73,74]:

(14) $$\begin{array}{c} S=-\mathrm{Tr}\left(\rho \log_{2}\rho \right)=-\sum_{i=1}^{n}\lambda^{\left(i\right)}\log_{2}\lambda^{\left(i\right)}, \end{array}$$

where $\lambda^{\left(i\right)}$ is *i*th eigenvalue of density matrix ρ. Unlike the negativity, the von Neumann entropy is not required to find the partial transpose of the density matrix except for applying the partial trace to obtain the density matrix of subsystems. We write out explicitly nonzero eigenvalues of GHZ state to calculate entropies in Table 1 but do not list those of W-class state because of complicated expressions.

**Figure 19:**
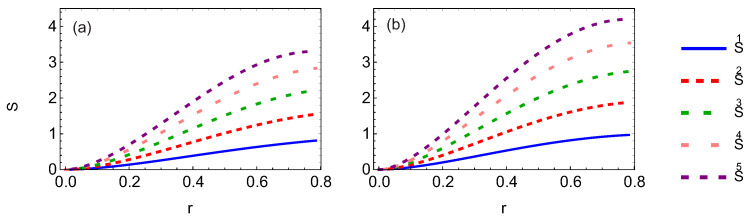
Panels (**a**,**b**) show the von Neumann entropy of the GHZ and W-class states when 1, 2, 3, 4, and all observers are accelerated.

Let us show the difference of the von Neumann entropy between GHZ and W-class states. As shown in Figure 19, entropy increases as the number of accelerated observers increases. This means that the system becomes more unstable. In the same condition, it is found that von Neumann entropy of the W-class state increases faster than that of GHZ.

## 4. Conclusions

In this work, we have studied the entanglement measures of pentapartite GHZ and W-class states by investigating the negativity and whole residual entanglement. We have carried out the cases when the 1, 2, 3, 4, or even all observers are accelerated. As we can see, the degree of entanglement will be degraded when the acceleration parameter *r* increases. However, we have verified again the fact that the degree of entanglement is dependent of the number of the accelerated particles. That is to say, the degree of the entanglement will decrease faster with the number of accelerated particles. The 1-4 and 2-3 tangles for both GHZ and W-class pentapartite states still exist even if the acceleration tends to infinity. Compared between GHZ and W-class states, we find that the degree of the entanglement of W-class state decreases faster than that of GHZ state when the accelerated parameter increases. The maximum values of the GHZ and W-class states are 1 and 0.8 without the acceleration. For 2-3 tangle case, we notice that when only one particle is accelerated, the GHZ entanglement decays faster than the W-class state, but with the increase of accelerated particles, the W-class entanglement decays faster than the GHZ state. However, we also note that the 2-3 tangle corresponding to the cases $N_{E_{1},ABCD}$$N_{D_{1}E_{1},ABC}$, $N_{D_{1}E_{1},ABC_{1}}$ and $N_{D_{1}E_{1},AB_{1}C_{1}}$ are always the smallest compared to other cases of the same type if we assume that the acceleration of particles always starts from the rightmost two in A(Alice), B(Bob), C(Charlie), D(David), E(Elly) qubits. The whole entanglement measurements show us that entanglement in GHZ state is greater than that of W-class state. However, we find that there is almost no difference between whole residual entanglements $\pi_{5}$ and $\Pi_{5}$. As far as the von Neumann entropy, compared with the tripartite and tetrapartite entangled systems, the von Neumann entropy of pentapartite system is larger than those of tripartite and tetrapartite cases. As the number of accelerated particles increases, the von Neumann entropy of the system increases accordingly. This implies that the system becomes more and more unstable with the increasing accelerated particles. Before ending this work, we give a useful remark on the difference of the negativity among the tripartite [43], tetrapartite [44], and present pentapartite cases. In the GHZ state case, all 1-1 tangles for them are equal to zero. For the W-class case, however, there only exists a common 1-1 tangle among them. Let us show their difference. For example, the $N_{\alpha ,\beta }$ of pentapartite, tetrapartite, and tripartite cases is equal to 0.12111, 0.2071, and 0.412023, respectively, at r=π/4, but $N_{\alpha_{1},\beta }$ of pentapartite and tetrapartite cases will disappear ($N_{\alpha_{1},\beta }=0$) at r>0.61548 and 0.785398, respectively, and $N_{\alpha_{1},\beta }=0.138071$ in the tripartite case at r=π/4. The $N_{\alpha_{1},\beta_{1}}$ of pentapartite, tetrapartite, and tripartite cases is equal to 0.38671, 0.472473, and 0.699185, respectively. This implies that the degree of the entanglement decays faster with the increasing entangled particles so that the system becomes more and more unstable.

## Figures and Tables

**Table 1:** Eigenvalues of GHZ density matrices in the noninertial frame.

Density Matrix	Eigenvalues
$\rho_{ABCDE_{I}}$	$\lambda_{31}=\frac{1}{4}\left(3+\cos\left(2r\right)\right)$ $\lambda_{32}=\frac{\sin^{2}\left(r\right)}{2}$
$\rho_{ABCD_{I}E_{I}}$	$\lambda_{29}=\frac{1}{16}\left(11+4\cos\left(2r\right)+\cos\left(4r\right)\right)$ $\lambda_{30}=\frac{\sin^{4}\left(r\right)}{2}$ $\lambda_{31}=\lambda_{32}=\frac{\sin^{2}\left(2r\right)}{8}$
$\rho_{ABC_{I}D_{I}E_{I}}$	$\lambda_{25}=\frac{1}{64}\left(15\cos\left(2r\right)+6\cos\left(4r\right)+\cos\left(6r\right)+42\right)$ $\lambda_{26}=\lambda_{27}=\lambda_{28}=\lambda_{29}=\lambda_{30}=\lambda_{31}=\frac{1}{2}\sin^{4}\left(r\right)\cos^{2}\left(r\right)$ $\lambda_{32}=\frac{\sin^{6}\left(r\right)}{2}$
$\rho_{AB_{I}C_{I}D_{I}E_{I}}$	$\lambda_{17}=\frac{1}{256}\left(56\cos\left(2r\right)+28\cos\left(4r\right)+8\cos\left(6r\right)+\cos\left(8r\right)+163\right)$ $\lambda_{18}=\lambda_{19}=\lambda_{20}=\lambda_{21}=\frac{1}{2}\sin^{2}\left(r\right)\cos^{6}\left(r\right)$ $\lambda_{22}=\lambda_{23}=\lambda_{24}=\lambda_{25}=\frac{1}{2}\sin^{6}\left(r\right)\cos^{2}\left(r\right)$ $\lambda_{26}=\frac{\sin^{8}\left(r\right)}{2}$ $\lambda_{27}=\lambda_{28}=\lambda_{29}=\lambda_{31}=\lambda_{32}=\frac{1}{32}\sin^{4}\left(2r\right)$
$\rho_{A_{I}B_{I}C_{I}D_{I}E_{I}}$	$\lambda_{1}=\lambda_{2}=\frac{1}{1024}(382+120\cos\left(4r\right)+10\cos\left(8r\right)+$ $-\sqrt{2}\sqrt{{\left(\cos\left(4r\right)+7\right)}^{2}\left(511\cos\left(4r\right)+62\cos\left(8r\right)+\cos\left(12r\right)+1474\right)})$ $\lambda_{3}=\lambda_{4}=\lambda_{5}=\lambda_{6}=\lambda_{7}=\lambda_{8}=\lambda_{9}=\lambda_{10}=\lambda_{11}=\lambda_{12}=\lambda_{13}=\lambda_{14}=\lambda_{15}=\lambda_{16}=\lambda_{17}=\frac{1}{2}\sin^{4}\left(r\right)\cos^{6}\left(r\right)$ $\lambda_{18}=\lambda_{19}=\lambda_{20}=\lambda_{21}=\lambda_{22}=\lambda_{23}=\lambda_{24}=\lambda_{25}=\lambda_{26}=\lambda_{27}=\frac{1}{2}\sin^{6}\left(r\right)\cos^{4}\left(r\right)$ $\lambda_{28}=\lambda_{29}=\lambda_{30}=\lambda_{31}=\lambda_{32}=\frac{1}{2}\sin^{8}\left(r\right)\cos^{2}\left(r\right)$

## Data Availability

The datasets generated during the current study are available from the corresponding author on reasonable request.

## Appendix A. Nonzero Elements of Density Matrices for GHZ and W-Class States in the Noninertial Frame

**Table A1:** Nonzero entries for GHZ density matrices.

Density Matrix	Nonzero Entries
$\rho_{ABCDE_{I}}$	$\left[1,1\right]=\left[2,2\right]=\frac{1}{2}\cos^{2}\left(r\right)$ $\left[32,1\right]=\left[1,32\right]=\frac{1}{2}\cos\left(r\right)$ $\left[32,32\right]=\frac{1}{2}$
$\rho_{ABCD_{I}E_{I}}$	$\left[1,1\right]=\frac{1}{2}\cos^{4}\left(r\right)$ $\left[32,1\right]=\left[1,32\right]=\frac{1}{2}\cos^{2}\left(r\right)$ $\left[2,2\right]=\left[3,3\right]=\frac{1}{8}\sin^{2}\left(2r\right)$ $\left[4,4\right]=\frac{1}{2}\sin^{4}\left(r\right)$ $\left[32,32\right]=\frac{1}{2}$
$\rho_{ABC_{I}D_{I}E_{I}}$	$\left[1,1\right]=\frac{1}{2}\cos^{6}\left(r\right)$ $\left[1,32\right]=\left[32,1\right]=\frac{1}{2}\cos^{3}\left(r\right)$ $\left[2,2\right]=\left[3,3\right]=\left[5,5\right]=\frac{1}{2}\cos^{4}\left(r\right)\sin^{2}\left(r\right)$ $\left[4,4\right]=\left[6,6\right]=\left[7,7\right]=\frac{1}{2}\cos^{2}\left(r\right)\sin^{4}\left(r\right)$ $\left[8,8\right]=\frac{1}{2}\sin^{6}\left(r\right)$ $\left[32,32\right]=\frac{1}{2}$
$\rho_{AB_{I}C_{I}D_{I}E_{I}}$	$\left[1,1\right]=\frac{1}{2}\cos^{8}\left(r\right)$ $\left[1,32\right]=\left[32,1\right]=\frac{1}{2}\cos^{4}\left(r\right)$ $\left[2,2\right]=\left[3,3\right]=\left[5,5\right]=\left[9,9\right]=\frac{1}{2}\cos^{6}\left(r\right)\sin^{2}\left(r\right)$ $\left[4,4\right]=\left[6,6\right]=\left[7,7\right]=\left[10,10\right]=\left[11,11\right]=\left[13,13\right]=\frac{1}{32}\sin^{4}\left(2r\right)$ $\left[8,8\right]=\left[12,12\right]=\left[14,14\right]=\left[15,15\right]=\frac{1}{2}\cos^{2}\left(r\right)\sin^{6}\left(r\right)$ $\left[16,16\right]=\frac{1}{2}\sin^{8}\left(r\right)$ $\left[32,32\right]=\frac{1}{2}$
$\rho_{A_{I}B_{I}C_{I}D_{I}E_{I}}$	$\left[1,1\right]=\frac{1}{2}\cos^{10}\left(r\right)$ $\left[1,32\right]=\left[32,1\right]=\frac{1}{2}\cos^{5}\left(r\right)$ $\left[2,2\right]=\left[3,3\right]=\left[5,5\right]=\left[9,9\right]=\left[17,17\right]=\frac{1}{2}\cos^{8}\left(r\right)\sin^{2}\left(r\right)$ $\left[13,13\right]=\left[4,4\right]=\left[19,19\right]=\left[10,10\right]=\left[11,11\right]=\left[6,6\right]=\left[18,18\right]=\left[21,21\right]=\left[25,25\right]=\left[7,7\right]=\frac{1}{2}\cos^{6}\left(r\right)\sin^{4}\left(r\right)$ $\left[8,8\right]=\left[12,12\right]=\left[14,14\right]=\left[15,15\right]=\left[20,20\right]=\left[22,22\right]=\left[23,23\right]=\left[26,26\right]=\left[27,27\right]=\left[29,29\right]=\frac{1}{2}\cos^{4}\left(r\right)\sin^{6}\left(r\right)$ $\left[16,16\right]=\left[24,24\right]=\left[28,28\right]=\left[30,30\right]=\left[31,31\right]=\frac{1}{2}\cos^{2}\left(r\right)\sin^{8}\left(r\right)$ $\left[32,32\right]=\frac{1}{2}+\frac{1}{2}\sin^{10}\left(r\right)$

**Table A2:** Nonzero entries for pentapartite W-class state.

Density Matrix	Nonzero Entries
$\rho_{{\mathrm{ABCDE}}_{1}}$	$\left[2,2\right]=\frac{1}{5}$ $\left[2,3\right]=\left[2,5\right]=\left[2,9\right]=\left[2,17\right]=\left[3,2\right]=\left[5,2\right]=\left[9,2\right]=\left[17,2\right]=\frac{1}{5}\cos\left(r\right)$ $\left[3,3\right]=\left[3,5\right]=\left[3,9\right]=\left[3,17\right]=\left[5,3\right]=\left[5,5\right]=\left[5,9\right]=\left[5,17\right]=\left[9,3\right]=\left[9,5\right]=\left[9,9\right]=\left[9,17\right]=\left[17,3\right]=\left[17,5\right]=\left[17,9\right]=\left[17,17\right]=\frac{1}{5}\cos^{2}\left(r\right)$ [4,4],[4,6]=[4,10]=[4,18]=[6,4]=[6,6]=[6,10]=[6,18]=[10,4]=[10,6]=[10,10]=[10,18]=[18,4]=[18,6]=[18,10]=[18,18] $=\frac{1}{5}\sin^{2}\left(r\right)$
$\rho_{{\mathrm{ABCD}}_{1}E_{1}}$	$\left[2,2\right]=\left[2,3\right]=\left[3,2\right]=\left[3,3\right]=\frac{1}{5}\cos^{2}\left(r\right)$ $\left[2,5\right]=\left[2,9\right]=\left[2,17\right]=\left[3,5\right]=\left[3,9\right]=\left[3,17\right]=\left[5,2\right]=\left[5,3\right]=\left[9,2\right]=\left[9,3\right]=\left[17,2\right]=\left[17,3\right]=\frac{1}{5}\cos^{3}\left(r\right)$ $\left[4,4\right]=\frac{2}{5}\sin^{2}\left(r\right)$ $\left[4,6\right]=\left[4,7\right]=\left[4,10\right]=\left[4,11\right]=\left[4,18\right]=\left[4,19\right]=\left[6,4\right]=\left[7,4\right]=\left[10,4\right]=\left[11,4\right]=\left[18,4\right]=\left[19,4\right]=\frac{1}{5}\sin^{2}\left(r\right)\cos\left(r\right)$ $\left[5,5\right]=\left[5,9\right]=\left[5,17\right]=\left[9,5\right]=\left[9,9\right]=\left[9,17\right]=\left[17,5\right]=\left[17,9\right]=\left[17,17\right]=\frac{1}{5}\cos^{4}\left(r\right)$ [6,6],[6,10]=[6,18]=[7,7]=[7,11]=[7,19]=[10,6]=[10,10]=[10,18]=[11,7]=[11,11]=[11,19]=[18,6]=[18,10]=[18,18]=[19,7]= $\left[19,11\right]=\left[19,19\right]=\frac{1}{20}\sin^{2}\left(2r\right)$ $\left[8,8\right]=\left[8,12\right]=\left[8,20\right]=\left[12,8\right]=\left[12,12\right]=\left[12,20\right]=\left[20,8\right]=\left[20,12\right]=\left[20,20\right]=\frac{1}{5}\sin^{4}\left(r\right)$
$\rho_{{\mathrm{ABC}}_{1}D_{1}E_{1}}$	$\left[2,2\right]=\left[2,3\right]=\left[2,5\right]=\left[3,2\right]=\left[3,3\right]=\left[3,5\right]=\left[5,2\right]=\left[5,3\right]=\left[5,5\right]=\frac{1}{5}\cos^{4}\left(r\right)$ $\left[2,9\right]=\left[2,17\right]=\left[3,9\right]=\left[3,17\right]=\left[5,9\right]=\left[5,17\right]=\left[9,2\right]=\left[9,3\right]=\left[9,5\right]=\left[17,2\right]=\left[17,3\right]=\left[17,5\right]=\frac{1}{5}\cos^{5}\left(r\right)$ $\left[4,4\right]=\left[6,6\right]=\left[7,7\right]=\frac{1}{10}\sin^{2}\left(2r\right)$ $\left[4,6\right]=\left[4,7\right]=\left[6,4\right]=\left[6,7\right]=\left[7,4\right]=\left[7,6\right]=\frac{1}{20}\sin^{2}\left(2r\right)$ [4,10]=[4,11]=[4,18]=[4,19]=[6,10]=[6,13]=[6,18]=[6,21]=[7,11]=[7,13]=[7,19]=[7,21]=[10,4]=[10,6]=[11,4]=[11,7]= $\left[13,6\right]=\left[13,7\right]=\left[18,4\right]=\left[18,6\right]=\left[19,4\right]=\left[19,7\right]=\left[21,6\right]=\left[21,7\right]=\frac{1}{5}\sin^{2}\left(r\right)\cos^{3}\left(r\right)$ $\left[8,8\right]=\frac{3}{5}\sin^{4}\left(r\right)$ $\left[8,12\right],\left[8,14\right]=\left[8,15\right]=\left[8,20\right]=\left[8,22\right]=\left[8,23\right]=\left[12,8\right]=\left[14,8\right]=\left[15,8\right]=\left[20,8\right]=\left[22,8\right]=\left[23,8\right]=\frac{1}{5}\sin^{4}\left(r\right)\cos\left(r\right)$ $\left[9,9\right]=\left[9,17\right]=\left[17,9\right]=\left[17,17\right]=\frac{1}{5}\cos^{6}\left(r\right)$ $\left[10,10\right],\left[10,18\right]=\left[11,11\right]=\left[11,19\right]=\left[13,13\right]=\left[13,21\right]=\left[18,10\right]=\left[18,18\right]=\left[19,11\right]=\left[19,19\right]=\left[21,13\right]=\left[21,21\right]=\frac{1}{5}\sin^{2}\left(r\right)\cos^{4}\left(r\right)$ $\left[12,12\right]=\left[12,20\right]=\left[14,14\right]=\left[14,22\right]=\left[15,15\right]=\left[15,23\right]=\left[20,12\right]=\left[20,20\right]=\left[22,14\right]=\left[22,22\right]=\left[23,15\right]=\left[23,23\right]=1/5\cos{\left(r\right)}^{2}\sin{\left(r\right)}^{4}$ $\left[16,16\right]=\left[16,24\right]=\left[24,16\right]=\left[24,24\right]=\frac{1}{5}\sin^{6}\left(r\right)$
$\rho_{{\mathrm{AB}}_{1}C_{1}D_{1}E_{1}}$	$\left[2,2\right]=\left[2,3\right]=\left[2,5\right]=\left[2,9\right]=\left[3,2\right]=\left[3,3\right]=\left[3,5\right]=\left[3,9\right]=\left[5,2\right]=\left[5,3\right]=\left[5,5\right]=\left[5,9\right]=\left[9,2\right]=\left[9,3\right]=\left[9,5\right]=\left[9,9\right]=\frac{1}{5}\cos^{6}\left(r\right)$ $\left[2,17\right]=\left[3,17\right]=\left[5,17\right]=\left[9,17\right]=\left[17,2\right]=\left[17,3\right]=\left[17,5\right]=\left[17,9\right]=\frac{1}{5}\cos^{7}\left(r\right)$ $\left[4,4\right]=\left[6,6\right]=\left[7,7\right]=\left[10,10\right]=\left[11,11\right]=\left[13,13\right]=\frac{2}{5}\sin^{2}\left(r\right)\cos^{4}\left(r\right)$ [4,6]=[4,7]=[4,10]=[4,11]=[6,4]=[6,7]=[6,10]=[6,13]=[7,4]=[7,6]=[7,11]=[7,13]=[10,4]=[10,6]=[10,11]=[10,13]=[11,4] $=\left[11,7\right]=\left[11,10\right]=\left[11,13\right]=\left[13,6\right]=\left[13,7\right]=\left[13,10\right]=\left[13,11\right]=\frac{1}{5}\sin^{2}\left(r\right)\cos^{4}\left(r\right)$ [4,18]=[4,19]=[6,18]=[6,21]=[7,19]=[7,21]=[10,18]=[10,25]=[11,19]=[11,25]=[13,21]=[13,25]=[18,4]=[18,6]=[18,10]= $\left[19,4\right]=\left[19,7\right]=\left[19,11\right]=\left[21,6\right]=\left[21,7\right]=\left[21,13\right]=\left[25,10\right]=\left[25,11\right]=\left[25,13\right]=\frac{1}{5}\sin^{2}\left(r\right)\cos^{5}\left(r\right)$ $\left[8,8\right]=\left[12,12\right]=\left[14,14\right]=\left[15,15\right]=\frac{3}{5}\sin^{4}\left(r\right)\cos^{2}\left(r\right)$ $\left[8,12\right]=\left[8,14\right]=\left[8,15\right]=\left[12,8\right]=\left[12,14\right]=\left[12,15\right]=\left[14,8\right]=\left[14,12\right]=\left[14,15\right]=\left[15,8\right]=\left[15,12\right]=\left[15,14\right]=\frac{1}{5}\sin^{4}\left(r\right)\cos^{2}\left(r\right)$ [8,20]=[8,22]=[8,23]=[12,20]=[12,26]=[12,27]=[14,22]=[14,26]=[14,29]=[15,23]=[15,27]=[15,29]=[20,8]=[20,12]=[22,8]= $\left[22,14\right]=\left[23,8\right]=\left[23,15\right]=\left[26,12\right]=\left[26,14\right]=\left[27,12\right]=\left[27,15\right]=\left[29,14\right]=\left[29,15\right]=\frac{1}{5}\sin^{4}\left(r\right)\cos^{3}\left(r\right)$ $\left[16,16\right]=\frac{4}{5}\sin^{6}\left(r\right)$ $\left[16,24\right]=\left[16,28\right]=\left[16,30\right]=\left[16,31\right]=\left[24,16\right]=\left[28,16\right]=\left[30,16\right]=\left[31,16\right]=\frac{1}{5}\sin^{6}\left(r\right)\cos\left(r\right)$ $\left[17,17\right]=\frac{1}{5}\cos^{8}\left(r\right)$ $\left[18,18\right]=\left[19,19\right]=\left[21,21\right]=\left[25,25\right]=\frac{1}{5}\sin^{2}\left(r\right)\cos^{6}\left(r\right)$ $\left[20,20\right]=\left[22,22\right]=\left[23,23\right]=\left[26,26\right]=\left[27,27\right]=\left[29,29\right]=\frac{1}{80}\sin^{4}\left(2r\right)$ $\left[24,24\right]=\left[28,28\right]=\left[30,30\right]=\left[31,31\right]=\frac{1}{5}\sin^{6}\left(r\right)\cos^{2}\left(r\right)$ $\left[32,32\right]=\frac{1}{5}\sin^{8}\left(r\right)$
$\rho_{A_{1}B_{1}C_{1}D_{1}E_{1}}$	[2,2]=[2,3]=[2,5]=[2,9]=[2,17]=[3,2]=[3,3]=[3,5]=[3,9]=[3,17]=[5,2]=[5,3]=[5,5]=[5,9]=[5,17]=[9,2]=[9,3]=[9,5]= $\left[9,9\right]=\left[9,17\right]=\left[17,2\right]=\left[17,3\right]=\left[17,5\right]=\left[17,9\right]=\left[17,17\right]=\frac{1}{5}\cos^{8}\left(r\right)$ $\left[4,4\right]=\left[6,6\right]=\left[7,7\right]=\left[10,10\right]=\left[11,11\right]=\left[13,13\right]=\left[18,18\right]=\left[19,19\right]=\left[21,21\right]=\left[25,25\right]=\frac{2}{5}\sin^{2}\left(r\right)\cos^{6}\left(r\right)$ [4,6]=[4,7]=[4,10]=[4,11]=[4,18]=[4,19]=[6,4]=[6,7]=[6,10]=[6,13]=[6,18]=[6,21]=[7,4]=[7,6]=[7,11]=[7,13]=[7,19]= [7,21]=[10,4]=[10,6]=[10,11]=[10,13]=[10,18]=[10,25]=[11,4]=[11,7]=[11,10]=[11,13]=[11,19]=[11,25]=[13,6]=[13,7]= [13,10]=[13,11]=[13,21]=[13,25]=[18,4]=[18,6]=[18,10]=[18,19]=[18,21]=[18,25]=[19,4]=[19,7]=[19,11]=[19,18]=[19,21]= $\left[19,25\right]=\left[21,6\right]=\left[21,7\right]=\left[21,13\right]=\left[21,18\right]=\left[21,19\right]=\left[21,25\right]=\left[25,10\right]=\left[25,11\right]=\left[25,13\right]=\left[25,18\right]=\left[25,19\right]=\left[25,21\right]=\frac{1}{5}\sin^{2}\left(r\right)\cos^{6}\left(r\right)$ $\left[8,8\right],\left[12,12\right]=\left[14,14\right]=\left[15,15\right]=\left[20,20\right]=\left[22,22\right]=\left[23,23\right]=\left[26,26\right]=\left[27,27\right]=\left[29,29\right]=\frac{3}{80}\sin^{4}\left(2r\right)$ [8,12]=[8,14]=[8,15]=[8,20]=[8,22]=[8,23]=[12,8]=[12,14]=[12,15]=[12,20]=[12,26]=[12,27]=[14,8]=[14,12]=[14,15]= [14,22]=[14,26]=[14,29]=[15,8]=[15,12]=[15,14]=[15,23]=[15,27]=[15,29]=[20,8]=[20,12]=[20,22]=[20,23]=[20,26]=[20,27] =[22,8]=[22,14]=[22,20]=[22,23]=[22,26]=[22,29]=[23,8]=[23,15]=[23,20]=[23,22]=[23,27]=[23,29]=[26,12]=[26,14]= [26,20]=[26,22]=[26,27]=[26,29]=[27,12]=[27,15]=[27,20]=[27,23]=[27,26]=[27,29]=[29,14]=[29,15]=[29,22]=[29,23]= $\left[29,26\right]=\left[29,27\right]=\frac{1}{80}\sin^{4}\left(2r\right)$ $\left[16,16\right]=\left[24,24\right]=\left[28,28\right]=\left[30,30\right]=\left[31,31\right]=\frac{4}{5}\sin^{6}\left(r\right)\cos^{2}\left(r\right)$ [16,24]=[16,28]=[16,30]=[16,31]=[24,16]=[24,28]=[24,30]=[24,31]=[28,16]=[28,24]=[28,30]=[28,31]=[30,16]=[30,24]= $\left[30,28\right]=\left[30,31\right]=\left[31,16\right]=\left[31,24\right]=\left[31,28\right]=\left[31,30\right]=\frac{1}{5}\sin^{6}\left(r\right)\cos^{2}\left(r\right)$ $\left[32,32\right]=\sin^{8}\left(r\right)$

## Appendix B. Analytical Expressions of 1-4 Tangles for GHZ and W-Class States

$$\begin{array}{c} \;\mathrm{For}\;\mathrm{GHZ}\;\mathrm{state}\;\mathrm{case}:\\ N_{E_{I},\mathrm{ABCD}}=\cos^{2}\left(r\right)\\ N_{D,\mathrm{ABC}E_{I}}=N_{C,\mathrm{ABD}E_{I}}=N_{B,\mathrm{ACD}E_{I}}=N_{A,\mathrm{BCD}E_{I}}=\cos\left(r\right)\\ N_{E_{I},{\mathrm{ABCD}}_{I}}=N_{D_{I},{\mathrm{ABCE}}_{I}}=\frac{1}{16}\left\{-1+\cos\left(4r\right)+2\sqrt{2}\cos^{2}\left(r\right)\sqrt{35-4\cos(2r)+\cos(4r)}\right\}\\ N_{C,{\mathrm{ABD}}_{I}E_{I}}=N_{B,{\mathrm{ACD}}_{I}E_{I}}=N_{A,{\mathrm{BCD}}_{I}E_{I}}=\cos^{2}\left(r\right)\\ N_{E_{I},{\mathrm{ABC}}_{I}D_{I}}=\frac{1}{64}\left\{-2-\cos\left(2r\right)+2\cos\left(4r\right)+\cos\left(6r\right)+4\sqrt{2}\cos^{3}\left(r\right)\sqrt{130-\cos(2r)-2\cos(4r)+\cos(6r)}\right\}\\ N_{B,{\mathrm{AC}}_{I}D_{I}E_{I}}=N_{A,{\mathrm{BC}}_{I}D_{I}E_{I}}=\cos^{3}\left(r\right)\\ N_{E_{I},{\mathrm{AB}}_{I}C_{I}D_{I}}=\frac{1}{256}\left\{-5-4\cos\left(2r\right)+4\cos\left(4r\right)+4\cos\left(6r\right)+\cos\left(8r\right)+8\sqrt{2}\cos^{4}\left(r\right)\sqrt{515-4\cos(4r)+\cos(8r)}\right\}\\ N_{A,B_{1}C_{1}D_{1}E_{1}}=\frac{1}{256}\{56\cos\left(2r\right)-28\cos\left(4r\right)+8\cos\left(6r\right)-\cos\left(8r\right)+\frac{1}{\sqrt{2}}[45904\cos\left(2r\right)+36680\cos\left(4r\right)+3824\cos\left(6r\right)\\ \;\;\;\;\;\;\;\;\;\;\;\;\;\;\;\;\;\;\;\;+2844\cos\left(8r\right)-560\cos\left(10r\right)+120\cos\left(12r\right)-16\cos\left(14r\right)+\cos\left(16r\right)+42275{]}^{1/2}-35\}\\ N_{E_{I},A_{I}B_{I}C_{I}D_{I}}=\frac{1}{256}\{-7+4\cos\left(4r\right)+3\cos\left(8r\right)+\sqrt{2}\cos^{2}\left(r\right)[11851+12532\cos\left(2r\right)+8156\cos\left(4r\right)-116\cos\left(6r\right)\\ {\;\;\;\;\;\;\;\;\;\;\;\;\;\;\;\;\;\;\;\;\;+436\cos\left(8r\right)-124\cos\left(10r\right)+36\cos\left(12r\right)-4\cos\left(14r\right)+\cos\left(16r\right)]}^{1/2}\}\;\\ \; \end{array}$$

$$\begin{array}{c} \mathrm{For}\;W-\mathrm{class}\;\mathrm{state}\;\mathrm{case}:\\ N_{E_{I},\mathrm{ABCD}}=\frac{1}{5}\left(2\cos\left(2r\right)+\sqrt{2}\sqrt{\cos(4r)+7}-2\right)\\ N_{D,\mathrm{ABC}E_{I}}=N_{C,\mathrm{ABD}E_{I}}=N_{B,\mathrm{ACD}E_{I}}=N_{A,\mathrm{BCD}E_{I}}=\\ \;\;\;\;\;\;\;\;\;\;\;\;\;\;\frac{1}{5}\left(-2\right)\mathrm{Root}\left[32\#1^{3}+\#1^{2}\left(16\cos\left(2r\right)-16\right)+\#1\left(-64\cos\left(2r\right)-12\cos\left(4r\right)-52\right)+\right.\\ \;\;\;\;\;\;\;\;\;\;\;\;\;\left.+3\cos(2r)-6\cos(4r)-3\cos(6r)+6\&,1\right]\\ N_{E_{I},{\mathrm{ABCD}}_{I}}=N_{D_{I},{\mathrm{ABCE}}_{I}}=\frac{1}{10}\mathrm{Root}\left[2\#1^{3}+\#1^{2}\left(8\cos\left(2r\right)+3\cos\left(4r\right)-11\right)+\#1(-64\cos\left(2r\right)+\right.\\ \;\;\;\;\;\;\;\;\;\;\;\;\;\;\left.-28\cos(4r)-36)+12\cos(2r)-12\cos(4r)-12\cos(6r)-3\cos(8r)+15\&,1\right]\\ N_{C,{\mathrm{ABD}}_{I}E_{I}}=N_{B,{\mathrm{ACD}}_{I}E_{I}}=N_{A,{\mathrm{BCD}}_{I}E_{I}}=\frac{1}{10}\mathrm{Root}\left[16\#1^{3}+\#1^{2}\left(8\cos\left(4r\right)-8\right)+\right.\\ \;\;\;\;\;\;\;\;\;\;\;\;\;\;\;\;\;\left.+\#1(-464\cos(2r)-208\cos(4r)-48\cos(6r)-4\cos(8r)-300)+48\cos(2r)+\right.\\ \;\;\;\;\;\;\;\;\;\;\;\;\;\;\;\;\;\left.-15\cos(4r)-40\cos(6r)-26\cos(8r)-8\cos(10r)-\cos(12r)+42\&,1\right]\\ N_{E_{I},{\mathrm{ABC}}_{I}D_{I}}=N_{D_{1},{\mathrm{ABC}}_{1}E_{1}}=N_{C_{1},{\mathrm{ABD}}_{1}E_{1}}=\frac{1}{10}\left|\mathrm{Root}\left[64\#1^{3}+(-16\cos\left(2r\right)+128\cos\left(4r\right)+\right.\right.\\ \;\;\;\;\;\;\;\;\;\;\;\;\;\;\;\;\;\left.\left.+16\cos\left(6r\right)-128)\#1^{2}+(-1728\cos\left(2r\right)-960\cos\left(4r\right)-320\cos\left(6r\right)+\right.\right.\\ \;\;\;\;\;\;\;\;\;\;\;\;\;\;\;\;\;\left.\left.-48\cos(8r)-1040)\#1+165\cos(2r)-22\cos(4r)-121\cos(6r)-100\cos(8r)\right.\right.\\ \;\;\;\;\;\;\;\;\;\;\;\;\;\;\;\;\;\left.\left.-43\cos(10r)-10\cos(12r)-\cos(14r)+132\&,1\right]\right|\\ N_{B,{\mathrm{AC}}_{I}D_{I}E_{I}}=N_{A,{\mathrm{BC}}_{I}D_{I}E_{I}}=-\frac{1}{10}\mathrm{Root}\left[2048\#1^{3}+\#1^{2}(-256\cos\left(2r\right)+512\cos\left(4r\right)+\right.\\ \;\;\;\;\;\;\;\;\;\;\;\;\;\;\;\;\;\left.+256\cos(6r)-512)+\#1(-52992\cos(2r)-30960\cos(4r)-12160\cos(6r)+\right.\\ \;\;\;\;\;\;\;\;\;\;\;\;\;\;\;\;\;\left.-2976\cos(8r)-384\cos(10r)-16\cos(12r)-31584)+2002\cos(2r)+208\cos(4r)+\right.\\ \;\;\;\;\;\;\;\;\;\;\;\;\;\;\;\;\;\left.-1092\cos(6r)-1288\cos(8r)-820\cos(10r)-336\cos(12r)+\right.\\ \;\;\;\;\;\;\;\;\;\;\;\;\;\;\;\;\;\left.-89\cos(14r)-14\cos(16r)-\cos(18r)+1430\&,1\right]\\ N_{A,B_{1}C_{1}D_{1}E_{1}}=\frac{1}{640}\left(\left|4\cos(2r)-4\cos(4r)-4\cos(6r)-\cos(8r)+\right.\right.\\ \;\;\;\;\;\;\;\;\;\;\;\;\;\;\;\;\left.\left.-32\sqrt{2}\sqrt{\cos^{12}\left(r\right)\left(60\cos\left(2r\right)+\cos\left(4r\right)+67\right)}+5\right|+\left|240\cos(2r)+92\cos(4r)+\right.\right.\\ \;\;\;\;\;\;\;\;\;\;\;\;\;\;\;\;\left.\left.+16\cos(6r)+\cos(8r)+\right.\right.\\ \;\;\;\;\;\;\;\;\;\;\;\;\;\;\;\;\left.\left.-8\sqrt{2}\sqrt{\cos^{8}\left(r\right)\left(904\cos\left(2r\right)+156\cos\left(4r\right)+56\cos\left(6r\right)+\cos\left(8r\right)+931\right)}+163\right|\right)\\ N_{A_{1},B_{1}C_{1}D_{1}E_{1}}=N_{E_{I},A_{I}B_{I}C_{I}D_{I}}=N_{D_{1},{\mathrm{AB}}_{1}C_{1}E_{1}}=N_{C_{1},{\mathrm{AB}}_{1}D_{1}E_{1}}=N_{B_{1},{\mathrm{AC}}_{1}D_{1}E_{1}}=\\ \;\;\;\;\;\;\;\;\;\;\;\;\;\;\frac{1}{640}\left|20\cos(2r)-20\cos(4r)-20\cos(6r)-5\cos(8r)+\right.\\ \;\;\;\;\;\;\;\;\;\;\;\;\;\;\left.-32\sqrt{2}\sqrt{\cos^{12}\left(r\right)\left(41\left(\cos\left(4r\right)+3\right)-36\cos\left(2r\right)\right)}+25\right| \end{array}$$

## Appendix C. Analytical Expressions of 2-3 Tangles for GHZ and W-Class States

$$\begin{array}{c} \;\mathrm{For}\;\mathrm{GHZ}\;\mathrm{state}\;\mathrm{case}:\\ N_{\mathrm{AB},{\mathrm{CDE}}_{I}}=N_{\mathrm{AD},{\mathrm{BCE}}_{I}}=N_{\mathrm{CD},{\mathrm{ABE}}_{I}}=N_{{\mathrm{BE}}_{I},\mathrm{ACD}}=N_{{\mathrm{AE}}_{I},\mathrm{BCD}}=\cos\left(r\right)\\ N_{\mathrm{AB},{\mathrm{CD}}_{I}E_{I}}=N_{{\mathrm{CE}}_{I},{\mathrm{ABD}}_{I}}=N_{{\mathrm{AE}}_{I},{\mathrm{BCD}}_{I}}=N_{{\mathrm{BD}}_{I},{\mathrm{ACE}}_{I}}=\cos^{2}\left(r\right)\\ N_{D_{I}E_{I},\mathrm{ABC}}=\frac{1}{16}\left\{-3+4\cos\left(2r\right)-\cos\left[4r\right]+\frac{\sqrt{227+200\cos(2r)+92\cos(4r)-8\cos(6r)+\cos(8r)}}{\sqrt{2}}\right\}\\ N_{\mathrm{AB},C_{I}D_{I}E_{I}}=\frac{1}{64}\{-10+15\cos\left(2r\right)-6\cos\left(4r\right)+\cos\left(6r\right)+\frac{1}{\sqrt{2}}[3022+3048\cos\left(2r\right)+2031\cos\left(4r\right)\\ \;\;\;\;\;\;\;\;\;\;\;\;\;\;\;\;\;\;\;\;\;+36\cos\left(6r\right)+66\cos\left(8r\right)-12\cos\left(10r\right)+\cos\left(12r\right){]}^{1/2}\}\\ N_{{\mathrm{AE}}_{I},{\mathrm{BC}}_{I}D_{I}}=N_{{\mathrm{BE}}_{I},{\mathrm{AC}}_{I}D_{I}}=\cos^{3}\left(r\right)\\ N_{D_{I}E_{I},{\mathrm{ABC}}_{I}}=\frac{1}{64}\{-2+\cos\left(2r\right)+2\cos\left(4r\right)-\cos\left(6r\right)\\ \;\;\;\;\;\;\;\;\;\;\;\;\;\;\;\;\;\;\;\;\;+2\sqrt{2}\cos^{2}\left(r\right)\sqrt{291+200\cos(2r)+28\cos(4r)-8\cos(6r)+\cos(8r)}\}\\ N_{{\mathrm{AB}}_{I},C_{I}D_{I}E_{I}}=\frac{1}{256}\{-5+4\cos\left(2r\right)+4\cos\left(4r\right)-4\cos\left(6r\right)+\cos\left(8r\right)+2\sqrt{2}\cos^{2}\left(r\right)[3534+3304\cos\left(2r\right)\\ \;\;\;\;\;\;\;\;\;\;\;\;\;\;\;\;\;\;\;\;+1519\cos\left(4r\right)-220\cos\left(6r\right)+66\cos\left(8r\right)-12\cos\left(10r\right)+\cos\left(12r\right){]}^{1/2}\}\\ N_{B_{I}E_{I},AC_{I}D_{I}}=\frac{1}{256}\left\{-3+4\cos\left(4r\right)-\cos\left(8r\right)+8\sqrt{2}\cos^{4}\left(r\right)\sqrt{547-56\cos(2r)+28\cos(4r)-8\cos(6r)+\cos(8r)}\right\}\\ N_{A_{I}B_{I},C_{I}D_{I}E_{I}}=\frac{1}{256}\{-3+4\cos\left(4r\right)-\cos\left(8r\right)+4\sqrt{2}\cos^{4}\left(r\right)[1122+848\cos\left(2r\right)+127\cos\left(4r\right)\\ {\;\;\;\;\;\;\;\;\;\;\;\;\;\;\;\;\;\;\;\;\;\;\;-72\cos\left(6r\right)+30\cos\left(8r\right)-8\cos\left(10r\right)+\cos\left(12r\right)]}^{1/2}\} \end{array}$$

$$\begin{array}{c} \;\mathrm{For}\;W-\mathrm{class}\;\mathrm{state}\;\mathrm{case}:\\ N_{\mathrm{AB},{\mathrm{CDE}}_{I}}=N_{\mathrm{AD},{\mathrm{BCE}}_{I}}=N_{\mathrm{CD},{\mathrm{ABE}}_{I}}=\frac{1}{5}\left(-2\right)\mathrm{Root}\left[4\#1^{3}+\#1^{2}\left(4\cos\left(2r\right)-4\right)+\right.\\ \;\;\;\;\;\;\;\;\;\left.\#1(-12\cos(2r)-2\cos(4r)-10)+\cos(2r)-2\cos(4r)-\cos(6r)+2\&,1\right]\\ N_{{\mathrm{BE}}_{I},\mathrm{ACD}}=N_{{\mathrm{AE}}_{I},\mathrm{BCD}}=\frac{1}{5}\left(-2\right)\mathrm{Root}\left[32\#1^{3}+\#1^{2}\left(48\cos\left(2r\right)-48\right)+\right.\\ \;\;\;\;\;\;\;\;\;\left.\#1(-96\cos(2r)-12\cos(4r)-84)+9\cos(2r)-18\cos(4r)-9\cos(6r)+18\&,1\right]\\ N_{\mathrm{AB},{\mathrm{CD}}_{I}E_{I}}=\frac{1}{10}\mathrm{Root}\left[8\#1^{3}+\#1^{2}\left(8\cos\left(4r\right)-8\right)+\#1(-352\cos\left(2r\right)+\right.\\ \;\;\;\;\;\;\;\;\;\left.-152\cos(4r)-32\cos(6r)-2\cos(8r)-230)+48\cos(2r)-15\cos(4r)+\right.\\ \;\;\;\;\;\;\;\;\;\left.-40\cos(6r)-26\cos(8r)-8\cos(10r)-\cos(12r)+42\&,1\right]\\ N_{{\mathrm{CE}}_{I},{\mathrm{ABD}}_{I}}=N_{{\mathrm{AE}}_{I},{\mathrm{BCD}}_{I}}=N_{{\mathrm{BD}}_{I},{\mathrm{ACE}}_{I}}=-\frac{1}{10}\mathrm{Root}\left[256\#1^{6}+\#1^{5}(1024\cos\left(2r\right)+\right.\\ \;\;\;\;\;\;\;\;\;\left.+384\cos\left(4r\right)-1408)+\#1^{4}\left(-8192\cos\left(2r\right)-7424\cos\left(4r\right)-64\cos\left(8r\right)-8896\right)+\right.\\ \;\;\;\;\;\;\;\;\;\left.\#1^{3}(5120\cos\left(2r\right)-5024\cos\left(4r\right)-4864\cos\left(6r\right)-4416\cos\left(8r\right)-256\cos\left(10r\right)+\right.\\ \;\;\;\;\;\;\;\;\;\left.-96\cos\left(12r\right)+9536)+\#1^{2}(-2048\cos\left(2r\right)+2432\cos\left(4r\right)+3072\cos\left(6r\right)+\right.\\ \;\;\;\;\;\;\;\;\;\left.+1440\cos(8r)-1024\cos(10r)-896\cos(12r)-8\cos(16r)-2968)+\right.\\ \;\;\;\;\;\;\;\;\;\left.+\#1(224\cos(2r)+3052\cos(4r)-448\cos(6r)-1616\cos(8r)+320\cos(10r)+\right.\\ \;\;\;\;\;\;\;\;\;\left.+526\cos(12r)-112\cos(14r)-92\cos(16r)+16\cos(18r)+6\cos(20r)-1876)+\right.\\ \;\;\;\;\;\;\;\;\;\left.-792\cos(4r)+495\cos(8r)-220\cos(12r)+66\cos(16r)-12\cos(20r)+\cos(24r)+462\&,1\right]\\ N_{D_{I}E_{I},\mathrm{ABC}}=\frac{1}{40}\left(2\sqrt{6}\sqrt{\cos^{4}\left(r\right)\left(20\cos\left(2r\right)+3\cos\left(4r\right)+41\right)}+3\cos\left(4r\right)-3\right)\\ N_{\mathrm{AB},C_{I}D_{I}E_{I}}=\frac{1}{80}\left|\cos\left(2r\right)-2\cos\left(4r\right)-\cos\left(6r\right)-8\sqrt{2}\sqrt{\cos^{8}\left(r\right)\left(20\cos\left(2r\right)+\cos\left(4r\right)+27\right)}+2\right|\\ N_{{\mathrm{AE}}_{I},{\mathrm{BC}}_{I}D_{I}}=N_{{\mathrm{BE}}_{I},{\mathrm{AC}}_{I}D_{I}}=-\frac{1}{10}\mathrm{Root}\left[134217728\#1^{7}+\#1^{6}(218103808\cos\left(2r\right)+\right.\\ \;\;\;\;\;\;\;\;\;\;\;\;\;\;\;\;\;\;\;\;\;\left.+167772160\cos\left(4r\right)+50331648\cos\left(6r\right)-436207616)+\#1^{5}(-5385486336\cos\left(2r\right)+\right.\\ \;\;\;\;\;\;\;\;\;\;\;\;\;\;\;\;\;\;\;\;\;+\left.-3138387968\cos(4r)-1048576000\cos(6r)-224395264\cos(8r)+\right.\\ \;\;\;\;\;\;\;\;\;\;\;\;\;\;\;\;\;\;\;\;\;\left.-8388608\cos\left(10r\right)+1048576\cos\left(12r\right)-3080716288)+\#1^{4}(2032795648\cos\left(2r\right)+\right.\\ \;\;\;\;\;\;\;\;\;\;\;\;\;\;\;\;\;\;\;\;\;\left.-248512512\cos(4r)-1369702400\cos(6r)-1225261056\cos(8r)+\right.\\ \;\;\;\;\;\;\;\;\;\;\;\;\;\;\;\;\;\;\;\;\;\left.-619970560\cos(10r)-196083712\cos(12r)-42795008\cos(14r)+\right.\\ \;\;\;\;\;\;\;\;\;\;\;\;\;\;\;\;\;\;\;\;\;\left.-4063232\cos\left(16r\right)-327680\cos\left(18r\right)+1673920512)+\#1^{3}(-312737792\cos\left(2r\right)+\right.\\ \;\;\;\;\;\;\;\;\;\;\;\;\;\;\;\;\;\;\;\;\;\left.+157515776\cos(4r)+343670784\cos(6r)+234180608\cos(8r)+\right.\\ \;\;\;\;\;\;\;\;\;\;\;\;\;\;\;\;\;\;\;\;\;\left.+42336256\cos(10r)-67977216\cos(12r)-65667072\cos(14r)+\right.\\ \;\;\;\;\;\;\;\;\;\left.-27828224\cos(16r)-7471104\cos(18r)-1458176\cos(20r)-131072\cos(22r)+\right.\\ \;\;\;\;\;\;\;\;\;\left.-20480\cos\left(24r\right)-294412288)+\#1^{2}(-17313024\cos\left(2r\right)+62495232\cos\left(4r\right)+\right.\\ \;\;\;\;\;\;\;\;\;\left.+36309248\cos(6r)-27261952\cos(8r)-28723456\cos(10r)+3595776\cos(12r)+\right.\\ \;\;\;\;\;\;\;\;\;\left.+11975936\cos(14r)+2299904\cos(16r)-2281728\cos(18r)-1091072\cos(20r)+\right.\\ \;\;\;\;\;\;\;\;\;\left.-21248\cos(22r)+113664\cos(24r)+54016\cos(26r)+11776\cos(28r)+\right.\\ \;\;\;\;\;\;\;\;\;\left.+256\cos(30r)-40163328)+\#1(3010304\cos(2r)-12497760\cos(4r)+\right.\\ \;\;\;\;\;\;\;\;\;\left.-6544384\cos(6r)+6131968\cos(8r)+5596160\cos(10r)-1353536\cos(12r)+\right.\\ \;\;\;\;\;\;\;\;\;\left.-2677248\cos(14r)-259712\cos(16r)+665088\cos(18r)+228800\cos(20r)+\right.\\ \;\;\;\;\;\;\;\;\;\left.-33792\cos(22r)-30976\cos(24r)-17408\cos(26r)-9040\cos(28r)+640\cos(30r)+\right.\\ \;\;\;\;\;\;\;\;\;\left.+2208\cos(32r)+640\cos(34r)+48\cos(36r)+7788000)-60996\cos(2r)+\right.\\ \;\;\;\;\;\;\;\;\;\left.+392496\cos(4r)+148954\cos(6r)-262548\cos(8r)-163710\cos(10r)+\right.\\ \;\;\;\;\;\;\;\;\;\left.128928\cos(12r)+121176\cos(14r)-41616\cos(16r)-64872\cos(18r)+\right.\\ \;\;\;\;\;\;\;\;\;\left.4896\cos(20r)+25245\cos(22r)+2958\cos(24r)-6903\cos(26r)-1944\cos(28r)+\right.\\ \;\;\;\;\;\;\;\;\;\left.+1210\cos(30r)+564\cos(32r)-102\cos(34r)-88\cos(36r)-3\cos(38r)+\right.\\ \;\;\;\;\;\;\;\;\;\left.6\cos(40r)+\cos(42r)-223652\&,1\right]\\ N_{D_{I}E_{I},{\mathrm{ABC}}_{I}}=N_{C_{1}D_{1},{\mathrm{ABE}}_{1}}=-\frac{1}{10}\mathrm{Root}\left[64\#1^{3}+\#1^{2}(224\cos\left(2r\right)+32\cos\left(4r\right)+32\cos\left(6r\right)+\right.\\ \;\;\;\;\;\;\;\;\;\left.-288)+\#1(-2632\cos(2r)-1538\cos(4r)-436\cos(6r)-100\cos(8r)+\right.\\ \;\;\;\;\;\;\;\;\;\left.-4\cos(10r)+2\cos(12r)-1436)+690\cos(2r)-212\cos(4r)-538\cos(6r)+\right.\\ \;\;\;\;\;\;\;\;\;\left.-356\cos(8r)-142\cos(10r)-44\cos(12r)-10\cos(14r)-\cos(16r)+613\&,1\right] \end{array}$$

$$\begin{array}{c} N_{{\mathrm{AB}}_{I},C_{I}D_{I}E_{I}}=N_{{\mathrm{AD}}_{1},B_{1}C_{1}E_{1}}=\frac{1}{40}\left|\mathrm{Root}\left[2048\#1^{4}+(-4096\cos\left(2r\right)+10240\cos\left(4r\right)+\right.\right.\\ \;\;\;\;\;\;\;\;\;\;\;\left.\left.+4096\cos\left(6r\right)+512\cos\left(8r\right)-10752)\#1^{3}+(-1179648\cos\left(2r\right)+\right.\right.\\ \;\;\;\;\;\;\;\;\;\;\;\left.\left.-772736\cos(4r)-368640\cos(6r)-120896\cos(8r)-24576\cos(10r)-2432\cos(12r)+\right.\right.\\ \;\;\;\;\;\;\;\;\;\;\;\left.\left.+16\cos\left(16r\right)-676816)\#1^{2}+(1036320\cos\left(2r\right)+163200\cos\left(4r\right)+\right.\right.\\ \;\;\;\;\;\;\;\;\;\;\;\left.\left.-499872\cos(6r)-648192\cos(8r)-457296\cos(10r)-220224\cos(12r)+\right.\right.\\ \;\;\;\;\;\;\;\;\;\;\;\left.\left.-76016\cos(14r)-18688\cos(16r)-3120\cos(18r)-320\cos(20r)-16\cos(22r)+\right.\right.\\ \;\;\;\;\;\;\;\;\;\;\;\left.\left.+724224)\#1+15504\cos(2r)-22287\cos(4r)-27588\cos(6r)-3534\cos(8r)+\right.\right.\\ \;\;\;\;\;\;\;\;\;\;\;\left.\left.+13740\cos(10r)+10011\cos(12r)-168\cos(14r)-3732\cos(16r)-1896\cos(18r)+\right.\right.\\ \;\;\;\;\;\;\;\;\;\;\;\left.\left.-15\cos(20r)+372\cos(22r)+174\cos(24r)+36\cos(26r)+3\cos(28r)+19380\&,1\right]\right|\\ N_{B_{I}E_{I},AC_{I}D_{I}}=N_{D_{1}E_{1},{\mathrm{AB}}_{1}C_{1}}=N_{B_{1}C_{1},{\mathrm{AD}}_{1}E_{1}}=\frac{1}{40}\left|\mathrm{Root}\left[512\#1^{4}+(14336\cos\left(2r\right)+\right.\right.\\ \;\;\;\;\;\;\;\;\;\;\;\left.\left.-3584\cos\left(4r\right)+2048\cos\left(6r\right)+128\cos\left(8r\right)-12928)\#1^{3}+(-309888\cos\left(2r\right)+\right.\right.\\ \;\;\;\;\;\;\;\;\;\;\;\left.\left.-208864\cos(4r)-75648\cos(6r)-29072\cos(8r)-7808\cos(10r)-288\cos(12r)+\right.\right.\\ \;\;\;\;\;\;\;\;\;\;\;\left.\left.+128\cos\left(14r\right)+4\cos\left(16r\right)-154996)\#1^{2}+(145024\cos\left(2r\right)-23680\cos\left(4r\right)+\right.\right.\\ \;\;\;\;\;\;\;\;\;\;\;\left.\left.-107264\cos(6r)-68608\cos(8r)-25344\cos(10r)-17216\cos(12r)-11328\cos(14r)+\right.\right.\\ \;\;\;\;\;\;\;\;\;\;\;\left.-4352\cos(16r)-1088\cos(18r)-64\cos(20r)+113920)\#1-15184\cos(2r)+\right.\\ \;\;\;\;\;\;\;\;\;\;\;\left.\left.+39941\cos(4r)+28724\cos(6r)-10566\cos(8r)-17660\cos(10r)-2953\cos(12r)+\right.\right.\\ \;\;\;\;\;\;\;\;\;\;\;\left.\left.+3976\cos(14r)+2204\cos(16r)+328\cos(18r)-123\cos(20r)-164\cos(22r)+\right.\right.\\ \;\;\;\;\;\;\;\;\;\;\;\left.\left.-90\cos(24r)-20\cos(26r)-\cos(28r)-28412\&,1\right]\right|\\ N_{A_{I}B_{I},C_{I}D_{I}E_{I}}=N_{B_{1}D_{1},A_{1}C_{1}E_{1}}=N_{D_{1}E_{1},A_{1}B_{1}C_{1}}=\frac{1}{40}\left|\mathrm{Root}\left[512\#1^{3}+(256\cos\left(2r\right)+\right.\right.\\ \;\;\;\;\;\;\;\;\;\;\;\left.\left.+2816\cos\left(4r\right)-256\cos\left(6r\right)+704\cos\left(8r\right)-3520)\#1^{2}+(-274560\cos\left(2r\right)+\right.\right.\\ \;\;\;\;\;\;\;\;\;\;\;\left.\left.-197120\cos(4r)-104832\cos(6r)-41216\cos(8r)-13440\cos(10r)+\right.\right.\\ \;\;\;\;\;\;\;\;\;\;\;\left.\left.-3584\cos(12r)-384\cos(14r)+64\cos(16r)-151360)\#1+\right.\right.\\ \;\;\;\;\;\;\;\;\;\;\;\left.\left.+18360\cos(2r)-18360\cos(4r)-28152\cos(6r)-9639\cos(8r)+8100\cos(10r)+\right.\right.\\ \;\;\;\;\;\;\;\;\;\;\;\left.\left.+9684\cos(12r)+3132\cos(14r)-1026\cos(16r)-1332\cos(18r)-540\cos(20r)+\right.\right.\\ \;\;\;\;\;\;\;\;\;\;\;\left.\left.-108\cos(22r)-9\cos(24r)+19890\&,1\right]0em1em\right| \end{array}$$
